# Aberrations of biochemical indicators in amyotrophic lateral sclerosis: a systematic review and meta-analysis

**DOI:** 10.1186/s40035-020-00228-9

**Published:** 2021-01-08

**Authors:** Yangfan Cheng, Yongping Chen, Huifang Shang

**Affiliations:** 1 Department of Neurology, Laboratory of Neurodegenerative Disorders, Rare Disease Center, West China Hospital, Sichuan University, Chengdu, 610041 China; 2 National Clinical Research Center for Geriatric, Laboratory of Neurodegenerative Disorders, West China Hospital, Sichuan University, Chengdu, 610041 China

**Keywords:** Amyotrophic lateral sclerosis, Biochemical indicators, Energy homeostasis, Iron metabolism, Creatinine kinase

## Abstract

Accumulating evidence has suggested that the pathological changes in amyotrophic lateral sclerosis (ALS) are not only confined to the central nervous system but also occur in the peripheral circulating system. Here, we performed a meta-analysis based on the PubMed, EMBASE, EBSCO, and CNKI databases, to find out biochemical indicators associated with energy metabolism, iron homeostasis, and muscle injury that are altered in ALS patients and their correlations with ALS phenotypes. Forty-six studies covering 17 biochemical indicators, representing 5454 ALS patients and 7986 control subjects, were included in this meta-analysis. Four indicators, including fasting blood glucose level (weighted mean difference [WMD] = 0.13, 95% CI [0.06–0.21], *p* = 0.001), serum ferritin level (WMD = 63.42, 95% CI [48.12–78.73], *p* < 0.001), transferrin saturation coefficient level (WMD = 2.79, 95% CI [1.52–4.05], *p* < 0.001), and creatine kinase level (WMD = 80.29, 95% CI [32.90–127.67], *p* < 0.001), were significantly higher in the ALS patients, whereas the total iron-binding capacity (WMD = − 2.42, 95% CI [− 3.93, − 0.90], *p* = 0.002) was significantly lower in ALS patients than in the control subjects. In contrast, the other 12 candidates did not show significant differences between ALS patients and controls. Moreover, pooled hazard ratios (HR) showed significantly reduced survival (HR = 1.38, 95% CI [1.02–1.88], *p* = 0.039) of ALS patients with elevated serum ferritin levels. These findings suggest that abnormalities in energy metabolism and disruption of iron homeostasis are involved in the pathogenesis of ALS. In addition, the serum ferritin level is negatively associated with the overall survival of ALS patients.

**Supplementary Information:**

The online version contains supplementary material available at 10.1186/s40035-020-00228-9.

## Background

Amyotrophic lateral sclerosis (ALS) is a relentlessly progressive paralytic disease characterized by degeneration of upper and lower motor neurons, which occurs following insidious focal weakness and spreads to most skeletal muscles, including the diaphragm [1, 2]. To date, there is no curative treatment for ALS and most patients die within 5 years of disease onset due to respiratory paralysis [3]. The ALS etiology is unfortunately poorly understood.

A recent epidemiological study has shown that the mean age for typical ALS disease onset is 61.8 ± 3.8 years [4], whereas the diagnosis of ALS has been reported to be delayed for over 1 year due to the significant overlap of clinical manifestations with other conditions in the early stages of ALS [5]. Therefore, exploring robust biomarkers is essential for the diagnosis of ALS. Biochemical indices in the blood or cerebrospinal fluid (CSF) are readily available compared to the brain tissue; they are regarded as indicators, and may also be involved in the development of disease, thus having been studied widely. Accumulating evidence has suggested that some indicators associated with energy homeostasis, including glucose [6–8], lipid [9–11] and protein levels [12–14], are aberrant in ALS patients. Moreover, abnormal iron metabolism [15–17] and creatinine kinase [18–20] have been reported in ALS patients. However, these studies have not reached a consensus; thus, a systematic meta-analysis is needed to clarify the changes in biochemical indicators in ALS and make a better prognosis.

In this review, we set out to go over the literature to examine if the commonly reported clinical biochemical indicators, which include total cholesterol (TC), low-density lipoprotein (LDL), high-density lipoprotein (HDL), triglyceride (TG), fasting blood glucose (FBG), fasting insulin, glucose in CSF, total protein in CSF, CSF/serum albumin quotient (Qalb), serum albumin, serum total protein, ferritin, transferrin, iron, total iron-binding capacity (TIBC), transferrin saturation coefficient (TSC), and creatine kinase (CK), are aberrant in ALS patients, and analyze their associations with patient survival.

## Methods

### Literature search strategy and selection criteria

Systematic review and meta-analysis was performed according to the Preferred Reporting Items for Systematic Review and Meta-Analyses guidelines [21]. The PubMed, EMBASE, EBSCO and CNKI databases were systematically searched until January 2020, using search terms of “amyotrophic lateral sclerosis OR ALS OR Lou Gehrig’s disease” AND “cholesterol OR triglycerides OR lipid” for lipid metabolism, “fasting glucose OR blood glucose OR blood sugar OR glycated hemoglobin A OR HbA1c OR hyperglycemia OR hypoglycemia OR glycemic index” for glycometabolism, “albumin OR prealbumin OR pre-albumin OR globulin” for protein metabolism, “ferritin OR transferrin OR iron” for iron metabolism, and “creatine kinase OR CK” for muscle injury. All papers were reviewed for titles and abstracts. References of the relevant articles were also reviewed to identify eligible articles.

The inclusion criteria for eligible articles were as follows: (1) written in English or Chinese; (2) providing definite diagnostic criteria for ALS patients; (3) being a case-control study or a cohort study; (4) the control group included healthy controls or several other neurological disorders (ONDs); and (5) reporting quantitative values of blood biochemical indicators in case-control studies, hazard ratio (HR) with 95% confidence interval (CI), or figures or tables of survival analyses. The exclusion criteria were as follows: (1) studies in animals or cell lines; (2) duplicate reports or different papers sharing the same cohort; and (3) reviews, case reports, editorials, and abstracts for conferences.

### Data extraction

For each study included, the following items were extracted: publication year, author name, location of the trial, sample size, age and sex of participants (ALSs and controls), and case ascertainment. Furthermore, the mean and standard deviation (SD) of levels of biochemical indicators, and HR with 95% CI were also extracted. Data presented in different units or other expression forms were converted to conform to the requirements [22, 23].

### Statistical analysis

The mean and SD of biochemical indicator concentrations were used to generate the effect size (ES) calculated as the weighted mean difference (WMD), to compare the differences between ALS patients and controls. To eliminate the bias from healthy/disease control and demographics of the study populations, separate analyses were carried out to examine the findings more carefully. We used a pooled HR with 95% CI to evaluate the association of blood biochemical indicators that show significant difference between ALS patients and controls, with survival time. For papers that provided Kaplan-Meier survival analyses, we transformed the figure into a data sheet and used the log rank test to obtain the HR and 95% CI [24]. The *I*^2^ statistic and Cochrane Q test were used to analyze between-study heterogeneity [25]. If *I*^2^ < 50% and *p* > 0.1 in the Q test, which means no obvious heterogeneity, the fixed-effects model was used to calculate the pooled estimate. Otherwise, the random-effects model was used for data with substantial heterogeneity. Subgroup analysis and meta-regression were conducted to explore potential sources of heterogeneity. Publication bias was determined using the Egger test, as described previously [26]. To evaluate the stability, we also performed sensitivity analysis by omitting each study in turn. All data were analyzed using STATA (version 15.0) software. *p* < 0.05 was considered statistically significant.

## Results

### Characteristics of the included studies

**Fig. 1:**
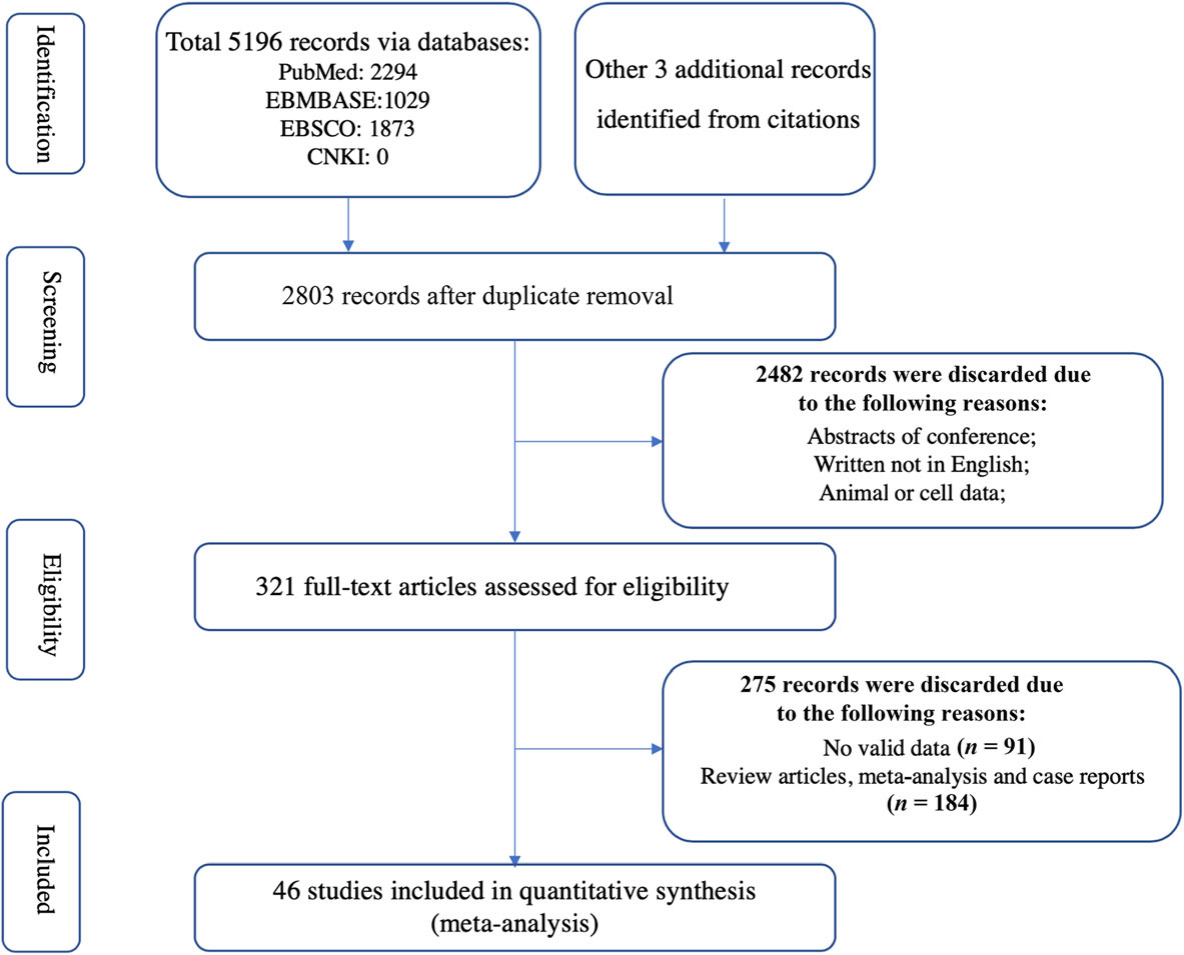
Flowchart of the literature search according to Preferred Reporting Items for Systematic Review and Meta-Analyses

As shown in Fig. 1, a systematic review of the literature identified 2803 papers from online databases. After screening titles and abstracts of these papers, 2482 papers were excluded, and 321 papers were further evaluated *via* a full-text review. After excluding articles without valid data, review articles, meta-analyses and case reports, 46 original articles were finally selected for the meta-analysis, which covered a total of 17 biochemical indicators in the serum or CSF in 5454 ALS patients and 7986 controls [8, 13, 16–18, 27–67]. Table S1 shows the characteristics of each study.

### Quantitative synthesis

**Fig. 2:**
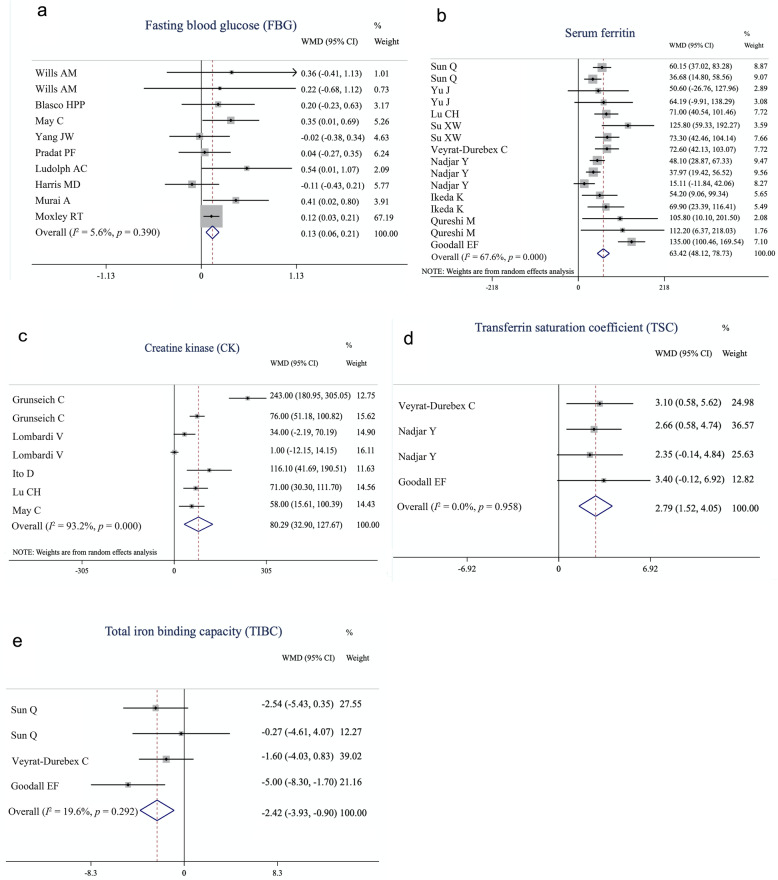
Forest plot showing the quantitative synthesis of fasting blood glucose (**a**), serum ferritin (**b**), creatine kinase (**c**), transferrin saturation coefficient (**d**) and total iron binding capacity (**e**) in amyotrophic lateral sclerosis patients and controls. Values and the corresponding 95% confidence intervals of individual studies are indicated by short solid lines. The weighted mean difference (WMD) and 95% confidence intervals are indicated by diamonds

**Fig. 3:**
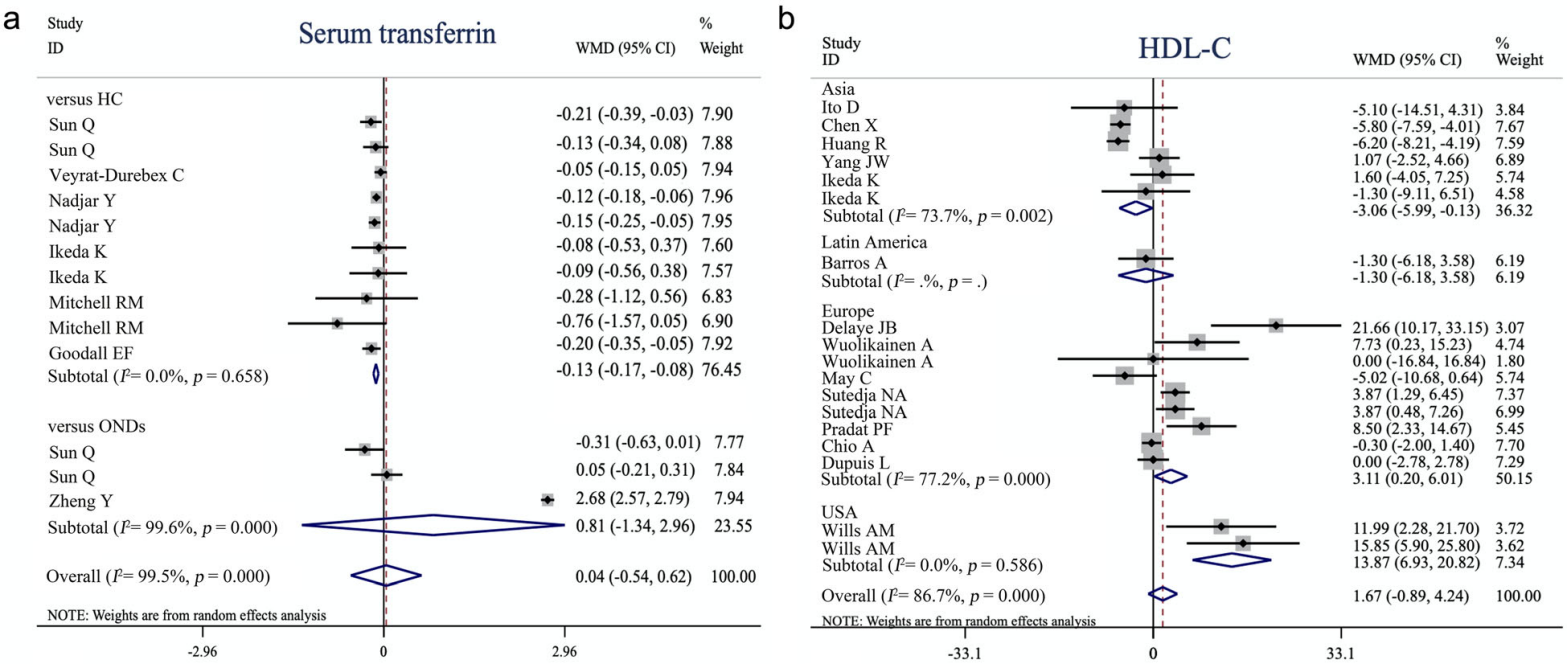
Forest plot showing separate analysis of serum transferrin based on control type (**a**) and high-density lipoprotein cholesterol based on ethnicity (**b**). Values and the corresponding 95% confidence intervals of individual studies are indicated by short solid lines. The weighted mean difference (WMD) and 95% confidence intervals are indicated by diamonds

As shown in the forest plot (Fig. 2), fixed-effects meta-analysis was performed to compare the FBG, TIBC, and TSC between ALS patients and controls, whereas for serum ferritin and CK, we applied a random-effects model. The levels of FBG (WMD = 0.13, 95% CI [0.06–0.21], *p* = 0.001), serum ferritin (WMD = 63.42, 95% CI [48.12–78.73], *p* < 0.001), TSC (WMD = 2.79, 95% CI [1.52–4.05], *p* < 0.001), and CK (WMD = 80.29, 95% CI [32.90–127.67], *p* < 0.001) were significantly higher in the ALS patients, whereas TIBC (WMD = − 2.42, 95% CI [− 3.93, − 0.90], *p* = 0.002) was significantly lower in the ALS patients, compared to the controls (Table 1). In contrast, no significant difference was found in lipid metabolism markers including TC, HDL-C, LDL-C, and TG, fasting serum insulin, serum albumin, serum total protein, serum transferrin, serum iron, CSF glucose, CSF total protein and Qalb. Considering that some ONDs *per se* are associated with peripheral biochemical changes, we conducted a separate analysis based on the control group to confirm our findings (Table 2). The serum transferrin level was significantly lower in ALS patients than in healthy controls (WMD = − 0.13, 95% CI [− 0.17, − 0.08], *p* < 0.001) but not significantly different from that in patients with ONDs (WMD = 0.81, 95% CI [− 1.34, 2.96], *p* = 0.46) (Fig. 3a). Furthermore, by performing a separate analysis based on ethnicity, we found ethnographic heterogeneity on HDL-C between patients with ALS and controls. HDL-C was lower in ALS patients in Asia (WMD = − 3.06, 95% CI [− 5.99, − 0.13], *p* = 0.041), but was significantly higher in the USA (WMD = 13.87, 95% CI [6.93–20.82], *p* < 0.001) and European studies (WMD = 3.11, 95% CI [0.20–6.01], *p* = 0.036) (Fig. 3b). Separate analyses of other indicators based on ethnicity are shown in Figure S1.

**Table 1:** Summary of comparative outcomes for measurements of biochemical indicators in blood and CSF

Biochemical indicator	No. of studies	No. of ALS/controls*	Main effect					Heterogeneity			Publication bias	
			WMD	Lower 95%CI	High 95% CI	z score	***p*** value	df	***p*** value	***I***^**2**^ statistic	Egger intercept	***p*** value
Total cholesterol	17	2801/4545	4.44	−2.62	11.50	1.23	0.22	21	< 0.001	89.40%	1.42	0.23
LDL-C	13	2677/4347	0.46	−6.56	7.47	0.13	0.90	16	< 0.001	95.10%	0.24	0.88
HDL-C	14	2707/4376	1.67	−0.89	4.24	1.28	0.20	17	< 0.001	86.70%	2.57	0.02
TG	11	2366/2261	0.11	−9.15	9.37	0.02	0.98	12	< 0.001	83.90%	−2.13	0.05
Fasting blood glucose	9	301/241	0.13	0.06	0.21	3.38	0.001	9	0.39	5.60%	0.51	0.35
Fasting insulin	6	94/88	−6.79	−29.27	15.70	0.59	0.55	6	< 0.001	89.70%	2.19	0.59
CSF glucose	3	132/91	0.04	−0.10	0.18	0.54	0.59	2	0.18	41.50%	1.91	0.56
CSF total protein	6	417/165	−1.53	−5.19	2.12	1.31	0.19	4	0.64	0.00%	−0.11	0.89
Qalb	7	627/212	0.44	−0.51	1.39	0.91	0.36	6	0.001	72.20%	−0.47	0.72
Serum albumin	3	630/611	−1.52	−5.53	2.48	0.74	0.46	2	< 0.001	94.80%	5.37	0.002
Serum total protein	3	705/690	−0.44	−1.03	0.14	1.49	0.14	2	< 0.001	98.80%	15.64	0.25
Serum ferritin	9	1661/1219	63.42	48.12	78.73	8.12	< 0.001	15	0.001	67.60%	1.89	0.06
Serum transferrin	7	1163/718	0.07	−0.58	0.72	0.21	0.83	10	< 0.001	99.60%	−0.41	0.96
Serum iron	6	974/659	0.34	−0.68	1.36	0.66	0.51	7	0.012	61.10%	−2.18	0.15
TIBC	3	236/304	−2.42	−3.93	−0.90	3.12	0.002	3	0.29	19.60%	0.05	0.99
TSC %	3	858/486	2.79	1.52	4.05	4.33	< 0.001	3	0.96	0	1.02	0.35
Creatine kinase	5	229/1843	80.29	32.90	127.67	3.32	0.001	6	< 0.001	93.20%	5.51	0.27

*Abbreviations*: *ALS* amyotrophic lateral sclerosis, *CSF* cerebrospinal fluid, *df* degrees of freedom, *HDL-C* high-density lipoprotein cholesterol, *LDL-C* low-density lipoprotein cholesterol, *NA* not available, *Qalb* CSF/serum albumin quotient, *TC* total cholesterol, *TG* triglyceride, *TIBC* total iron binding capacity, *TSC* transferrin saturation coefficient, *WMD* weighted mean difference. * controls include healthy controls and other neurological diseases controls

**Table 2:** Separate analyses based on control group for comparing biochemical indicator levels in blood and CSF

Biochemical indicator	vs HC						vs ONDs^a^					
	No. of studies	No. of ALS/controls	WMD	Lower 95%CI	High 95% CI	***p*** value	No. of studies	No. of ALS/controls	WMD	Lower 95%CI	High 95% CI	***p*** value
Total cholesterol	16	2762/4479	3.98	−3.48	11.44	0.30	1	39/66	10.87	−1.22	22.96	0.08
LDL-C	13	2677/4347	0.46	−6.56	7.47	0.90	0	NA	NA	NA	NA	NA
HDL-C	14	2707/4376	1.67	−0.89	4.24	0.20	0	NA	NA	NA	NA	NA
TG	11	2366/2261	0.11	−9.15	9.37	0.98	0	NA	NA	NA	NA	NA
Fasting blood glucose	8	220/204	0.13	0.05	0.21	0.001	1	81/37	0.20	−0.23	0.63	0.37
Fasting insulin	6	94/88	−6.79	−29.27	15.70	0.55	0	NA	NA	NA	NA	NA
CSF glucose	1	20/20	−0.05	−0.37	0.27	0.76	2	112/71	0.06	−0.10	0.21	0.45
CSF total protein^b^	0	NA	NA	NA	NA	NA	5	312/153	−1.45	−5.13	2.24	0.44
Qalb	1	14/20	2.00	−2.94	6.94	0.43	5	508/180	0.46	−0.55	1.47	0.37
Serum albumin	3	630/611	−1.52	−5.53	2.48	0.46	0	NA	NA	NA	NA	NA
Serum total protein	3	705/690	−0.44	−1.03	0.14	0.14	0	NA	NA	NA	NA	NA
Serum ferritin	9	1638/1217	63.43	48.12	78.73	< 0.001	2	572/255	58.72	29.23	88.21	< 0.001
Serum transferrin^c^	6	974/659	−0.13	−0.17	−0.08	< 0.001^a^	2	184/83	0.81	−1.34	2.96	0.46
Serum iron	6	974/659	0.34	−0.68	1.37	0.51	1	72/38	−0.49	−2.70	1.71	0.66
TIBC	3	236/304	−2.42	−3.93	−0.90	0.002	1	72/38	−1.74	−5.39	1.91	0.35
TSC %	3	858/486	2.79	1.52	4.05	< 0.001	0	NA	NA	NA	NA	NA
Creatine kinase	5	229/1843	80.29	32.90	127.67	0.001	0	NA	NA	NA	NA	NA

*Abbreviations*: *ALS* amyotrophic lateral sclerosis, *CI* confidence interval, *CSF* cerebrospinal fluid, *HC* healthy control, *HDL-C* high-density lipoprotein cholesterol, *LDL-C* low-density lipoprotein cholesterol, *NA* not available, *OND* other neurological disease, *Qalb* CSF/serum albumin quotient, *TG* triglyceride, *TIBC* total iron-binding capacity, *TSC%* transferrin saturation coefficient, *WMD* weighted mean difference

^a^: ONDs are other neurological diseases excluding ALS-related disease. Note: only one study compared ALS with lower motor neuron disease on total protein in CSF [Süssmuth. S. 2010]

^b^: Süssmuth. S et al. compared total protein in CSF and Qalb between ALS patients and lower motor neuron disease but the results did not have statistical significance (CSF total protein: WMD = −6.90, 95% CI [− 36.23–22.43], *p* = 0.65; Qalb: WMD = 2.00, 95% CI [−2.94–6.94], *p* = 0.47)

^c^: By performing a separate analysis based on control group, serum transferrin showed a significant decrease in ALS patients compared with healthy controls other than ONDs

### Investigation of heterogeneity

The heterogeneity analysis showed that the FBG, total protein and glucose in CSF, TIBC, and TSC% did not show heterogeneity among studies, while Qalb, serum ferritin, and serum iron presented substantial between-study heterogeneity, whereas evident heterogeneity among studies was shown for TC, LDL-C, HDL-C, TG, fasting insulin, serum albumin, serum total protein, serum transferrin, and CK (Table 1).

We then explored if there were potential variables that could explain the heterogeneity. As two indicators (serum ferritin *I*^2^ = 67.6%, *p* = 0.001; CK *I*^2^ = 93.2%, *p* < 0.001) that were of statistical importance between ALS patients and controls also showed evident heterogeneity between studies as described above, they were further subjected to a meta-regression and subgroup analysis.

Meta-regression analysis suggested that sex (male%) and the mean age of ALS patients did not have a moderate effect on the significant association between serum ferritin levels and ALS patients. Nevertheless, meta-regression of sample size showed a significant association between sample size and WMD for studies analyzing serum ferritin (*I*^2^_res = 39.79%, regression coefficient [SE] = − 0.10 [0.04], 95% CI [− 0.18, − 0.01], *p* = 0.03). Meanwhile, sex (male%), mean age of ALS patients, and disease duration did not affect the outcome of CK meta-analysis. Similarly, meta-regression of the sample size also showed a significant association between sample size and WMD for studies analyzing CK (*I*^2^_res = 80.53%, regression coefficient [SE] = 0.17 [0.05], 95% CI [0.05–0.28], *p* = 0.02).

**Fig. 4:**
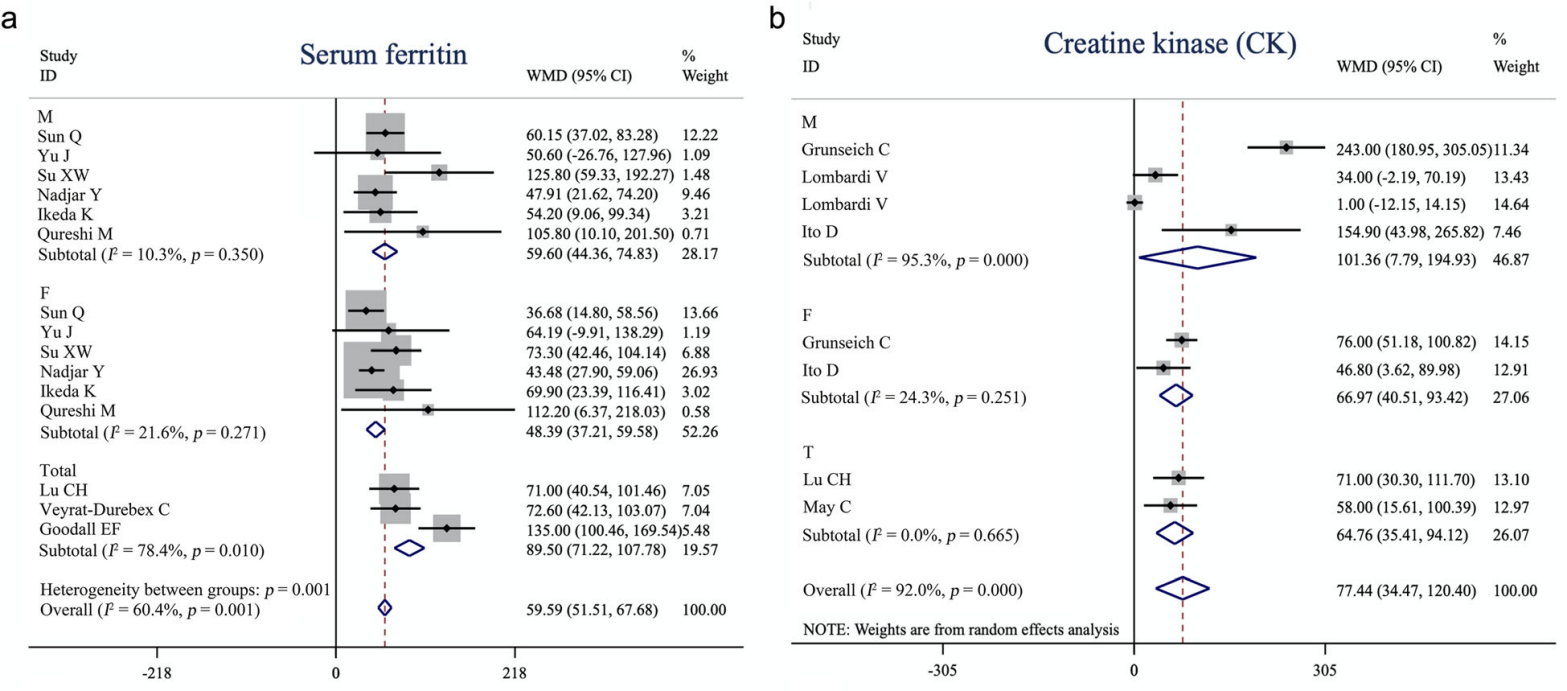
Forest plot showing subgroup analysis of serum ferritin (**a**) and creatine kinase (**b**) based on sex. M: male group, F: female group, Total: combined male and female patients. Values and the corresponding 95% confidence intervals of individual studies are indicated by short solid lines. The weighted mean difference (WMD) and 95% confidence intervals are indicated by diamonds

For serum ferritin, subgroup analysis based on sex (*n* = 6 studies) revealed no heterogeneity among studies either in males (Q = 5.57, *I*^2^ = 10.3%, *p* = 0.35) or in females (Q = 6.38, *I*^2^ = 21.6%, *p* = 0.271) (Fig. 4a), and ferritin was significantly elevated in both male (WMD = 59.60, 95% CI [44.36–74.83], *p* < 0.001) and female (WMD = 48.39, 95% CI [37.21–59.58], *p* < 0.001) ALS patients compared with that in sex-matched controls. In addition, after stratifying studies into the large sample group (sample size greater than average) and the small sample group (sample size less than the average), the heterogeneity was reduced to *I*^2^ < 50% (large sample group: Q = 13.25, *I*^2^ = 47.2%, *p* = 0.07; small sample group: Q = 11.37, *I*^2^ = 47.2%, *p* = 0.08). Both the large (*n* = 8, WMD = 53.85, 95% CI [45.07–62.64], *p* < 0.001) and small sample groups (*n* = 7, WMD = 59.59, 95% CI [51.51–67.68], *p* < 0.001) showed increased levels of serum ferritin in ALS patients. Compared with controls, there were significantly higher CK levels in male (*n* = 3, WMD = 101.36, 95% CI [7.79–194.93], *p* = 0.03) and female (*n* = 2, WMD = 66.97, 95% CI [40.51–93.42], *p* < 0.001) ALS patients. However, the between-study heterogeneity was increased in the male group (Q = 63.38, *I*^2^ = 95.3%, *p* < 0.001), and decreased in the female group (*n* = 2, Q = 1.32, *I*^2^ = 24.3%, *p* = 0.251) (Fig. 4b).

#### Potential publication bias assessment and sensitivity analysis

Sensitivity analysis demonstrated that the pooled WMDs of FBG, ferritin, TIBS, TBC%, and CK were stable, which indicated that the results were not affected by any single study. Furthermore, Egger’s test showed that there was no risk of publication bias among studies analyzing FBG, ferritin, TIBS, TBC%, and CK indicators in ALS (Table 1).

#### Association analysis

**Fig. 5:**
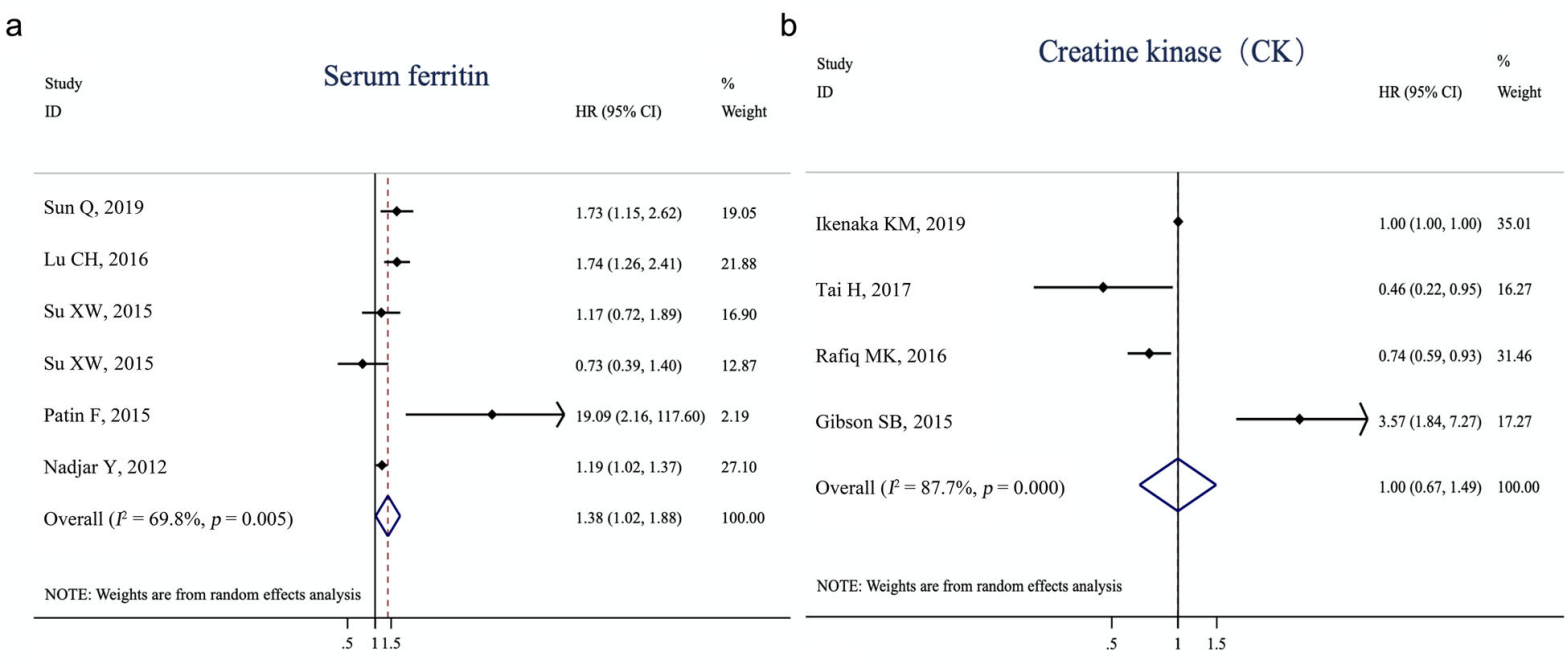
Forest plot showing associations of serum ferritin (**a**) and creatine kinase (**b**) levels with the overall survival in ALS patients. Pooled hazard ratios (HR) suggest significantly reduced survival in ALS patients with elevated serum ferritin, whereas creatine kinase levels did not play a significant role in the survival of ALS patients. Ratios and the corresponding 95% confidence intervals of studies are indicated by short solid lines. The averaged ratios and 95% confidence intervals are indicated by diamonds

Among the studies included in the meta-analysis, five studies underwent a survival analysis of serum ferritin levels and four studies underwent a survival analysis of CK levels in ALS patients. Given that their conclusions were not consistent, we then conducted a meta-analysis of the association of high vs. low levels of ferritin and CK with the overall survival of ALS patients. The associations between serum ferritin or CK levels and survival are shown in the forest plot (Fig. 5). The random-effects model was used because there was substantial heterogeneity among the studies related to ferritin (Q[df=5] = 16.56, *I*^2^ = 69.8%, *p* = 0.005). The pooled HR suggested significantly reduced survival (HR = 1.38, 95% CI [1.02–1.88], *p*= 0.04) of ALS patients with elevated serum ferritin levels, whereas CK levels did not affect the survival of patients with ALS (HR = 1.00, 95% CI [0.67–1.49], *p*= 0.99). No publication bias was identified in the Egger test (serum ferritin: Egger intercept = 1.35, *p* > 0.34; CK: Egger intercept = − 0.036, *p* > 0.87). The characteristics and summary statistics of studies included in the meta-analysis of survival are provided in Tables S2−S3.

The number of studies which showed precise biochemical values and survival analysis on FBG (*n* = 1), TSC (*n* = 0), and TIBC (*n* = 0) was not sufficient for meta-analysis; therefore, we did not perform meta-analysis on the associations of the three indicators with patient survival.

## Discussion

In this meta-analysis study, we compared 17 biochemical indicators between 5454 ALS patients and 7986 control subjects from 46 original studies, and found evidence of significantly higher FBG, ferritin, TSC and CK, and significantly lower TIBC in ALS patients than in controls. Only FBG presented a small ES (0 < ES < 1), while the other four indicators showed results associated with a large ES (ES > 1). In addition, within the five ALS-linked indicators, we found that the elevated serum ferritin levels were associated with reduced survival in patients with ALS. Sensitivity analysis indicated that the findings on FBG, ferritin, TIBC, TSC and CK in the ALS patients would not be affected by any single study included, suggesting the robustness of these results.

The difference in these clinical biochemical indicators between ALS patients and controls suggests that ALS is a multi-systemic disease with motor neuron degeneration, rather than a restricted central nervous system disorder [68]. Among the five ALS-associated indicators identified in the meta-analysis, three are related to iron metabolism. In addition, the serum transferrin level was significantly lower in ALS patients than in healthy controls, suggesting that the disruption of iron homeostasis is involved in the pathophysiological process of ALS [69]. Ferritin as a marker of body iron storage plays a role in iron sequestration where it functions as a ferroxidase, converting Fe^2+^ to Fe^3+^ as iron is internalized and sequestered in the ferritin mineral core [70, 71]. As ferritin can prevent iron from generating reactive oxygen species, it is considered to have an anti-oxidative cellular function [72, 73]. Under chronic inflammatory conditions, the ferritin levels are increased and TIBC, which is a measure of total serum transferrin (apotransferrin, monotransferrin, and diferric transferrin), could be decreased [74]. Therefore, the disruption of iron homeostasis might be the result of immune system activation [75], which is considered as one of the pathogeneses of ALS. TSC is often elevated in patients with *HFE*-linked hemochromatosis, while the H63D *HFE* gene polymorphism is considered as a risk factor for ALS [55, 76, 77]. Moreover, some imaging studies [78, 79] have suggested that ALS patients may be affected by iron overload. Our meta-analysis showed a negative association between serum ferritin levels and survival in ALS patients. Previous studies [80–82] have shown that the conservative iron chelation therapy may potentially serve as a treatment option to reduce iron accumulation and improve prognosis in ALS patients. Furthermore, our meta-analysis of survival data suggested the predictive role of serum ferritin in disease prognosis; thus, the ferritin level could be used as a tool to stratify patients in clinical trials. However, it remains to clarify whether the disruption of iron homeostasis is a cause or a consequence of ALS development, why ALS patients have increased iron body storage and how it relates to decreased survival. Further studies are needed to address these questions. The difference between the current meta-analysis and the previous one [83] was that we conducted a more comprehensive literature search that included more studies, with some inconsistent results, and that we analyzed the association between ALS patients and survival time and found a negative correlation between ferritin level and the overall survival.

Regarding the elevation of CK levels in ALS patients, Ito found that the serum CK was elevated not only in the early stages of ALS but also before its onset, achieved a maximum level around onset, and then declined after onset [18]. The pathophysiological mechanisms underlying this phenomenon might be the muscle cramp or active muscle denervation leading to the elevation of serum CK [84]. In addition, a study has reported denervation-induced membrane instability in muscle tissue and leakage of CK into the blood [85]. Hence, it is reasonable to suggest that the elevation of serum CK in ALS is caused by membrane instability or the destruction of muscle tissue due to the denervation and hyperexcitability of motor neurons [18]. Regarding the increase in FBG, there might be impaired control of fasting glucose levels and dysfunction of the glucose-insulin axis in patients with ALS. Considering the absence of beta cell dysfunction, fasting insulin levels and HbA1c did not significantly differ between ALS patients and controls, suggesting no evidence of insulin resistance [86], which is not consistent with our previous original study [6]. The potential mechanism underlying the elevation of FBG may be the chronic oxidative stress resulting from higher energy expenditure than intake in patients with ALS [68]. That is to say, some ALS patients with bulbar paralysis leading to dysphagia and an increase in resting energy expenditure [87–89] develop hypometabolism due to the denervation-linked muscle wasting. Long-term hypometabolism can activate chronic oxidative stress [90]. Therefore, the elevation of FBG levels is related to the oxidative stress, which also participates in the development of ALS. However, as described in our previous study [6], the FBG test is an excellent test for the “in the moment” glucose level, which, however, provides limited information on the trend of glucose level change over time. This is a limitation of our meta-analysis as there was not enough data on HbA1c or postprandial blood glucose hours after eating a meal, preventing us from evaluating changes in glucose level over time in ALS patients compared with that in controls. Regarding the interesting finding of ethnographic heterogeneity of HDL-C difference between ALS patients and controls, we suppose that the patient ethnicity may have an effect on the ALS phenotype. To our knowledge, this is the first report of the phenomenon of HDL-C. More research is needed to confirm this result.

Heterogeneity analysis using the *I*^2^ statistic and Cochrane Q test showed that the between-study heterogeneity of the 17 biochemical indicators varied from zero to high. Among the five indicators identified as associated with ALS, only serum ferritin and CK showed medium to high heterogeneity among studies. Therefore, we performed meta-regression analysis and subgroup analysis of these two indicators to explore confounders of the between-study heterogeneity. Meta-regression analysis suggested that the outcomes of both serum ferritin and CK might be influenced by the sample size, but not by other potential factors including sex (male%) and mean age of ALS patients. Due to the lack of complete data on disease duration in studies involving serum ferritin, we only conducted the meta-regression analysis on disease duration for CK, and found no influence of disease duration on the result. Therefore, the sample size of each study investigating serum ferritin and CK is one of the sources of heterogeneity, as partially confirmed by subgroup analysis of serum ferritin stratified by sample size. Subgroup analysis of serum ferritin stratified by sex not only reduced the heterogeneity between studies but also consistently revealed a significant association of ferritin with ALS, suggesting that sex was the second confounder of heterogeneity. Interestingly, Sun et al. [31] also found different distribution of serum ferritin in male and female patients with multiple system atrophy. For CK, despite the evident association in subgroup analysis based on sex, the between-study heterogeneity in male patients remained high, while the heterogeneity in female patients decreased significantly, suggesting that sex might be a confounder of between-study heterogeneity for CK.

There were some limitations in this study. First, some studies included in this analysis did not provide information on disease duration or disease status, which prevented us from performing a subgroup analysis of whether the differences in these indicators were associated with disease duration or severity. Second, we did not find indicators linked with energy metabolism in the CSF between patients with ALS and control subjects. Due to the hypothesis that CSF is a window to the brain, more biochemical indicators in the CSF should be investigated in further studies. Third, we did not perform meta-analysis on the associations of FBG, TSC and TIBC with survival due to the limited number of studies. Last, none of the biomarkers discussed in this meta-analysis are specific to ALS, and thus do not have potential use as diagnostic markers. Therefore, the indicators identified in our study should be confirmed in the future.

In conclusion, the findings of our meta-analysis revealed elevated FBG, ferritin, TSC, and CK levels and decreased TIBC in patients with ALS. In addition, the serum ferritin level was negatively associated with the overall survival of patients with ALS. These results provide further evidence that abnormalities in energy metabolism, disruption of iron homeostasis caused by oxidative stress, and abnormal immune activation participate in the pathophysiological process of ALS. Further studies are required to address whether these abnormalities are causes or consequences of ALS development and how they influence ALS. More studies are needed to translate the treatment potential of conservative iron chelation to benefit ALS patients.

## Supplementary Information

**Additional file 1: Table S1.** Characteristics of included studies measuring biochemical indicator levels in blood and CSF. **Table S2.** Characteristics and summary statistics of studies showing the association of serum ferritin with survival in ALS patients. **Table S3.** Characteristics and summary statistics of studies showing the association of creatine kinase with survival in ALS patients.

**Additional file 2: Fig. S1.** Forest plot showing separate analysis of total cholesterol, low-density lipoprotein cholesterol, triglyceride, fasting blood glucose, fasting insulin, CSF glucose, CSF total protein, Qalb, serum albumin, serum total protein, serum ferritin, serum transferrin, serum iron, total iron binding capacity, transferrin saturation coefficient and creatine kinase based on ethnicity, respectively. Values and the corresponding 95% confidence intervals of individual studies are indicated by short solid lines. The weighted mean difference (WMD) and 95% confidence intervals are indicated by diamonds.

## Data Availability

All data generated or analyzed during this study are included in this article and its supplementary materials.

## Abbreviations

ALS: Amyotrophic lateral sclerosis
CI: Confidence interval
CK: Creatine kinase
CSF: Cerebrospinal fluid
ES: Effective size
HDL: High-density lipoprotein
HR: Hazard ratios
LDL: Low-density lipoprotein
NDDs: Neurodegenerative diseases
Qalb: CSF/serum albumin quotient
SD: Standard deviation
TC: Total cholesterol
TG: Triglyceride
TIBC: Total iron-binding capacity
TSC: Transferrin saturation coefficient
WMD: Weighted mean difference
