# Supplementary material for: Aberrations of biochemical indicators in amyotrophic lateral sclerosis: a systematic review and meta-analysis
Source: Transl Neurodegener. 2021 Jan 8;10:3. doi: 10.1186/s40035-020-00228-9 (PMC7792103; doi:10.1186/s40035-020-00228-9)
Supplement: Supplementary file 1 — Additional file 1: Table S1. Characteristics of included studies measuring biochemical indicator levels in blood and CSF. Table S2. Characteristics and summary statistics of studies showing the association of serum ferritin with survival in ALS patients. Table S3. Characteristics and summary statistics of studies showing the association of creatine kinase with survival in ALS patients. [file 40035_2020_228_MOESM1_ESM.docx]

**Supplementary Table 1: Characteristics of included studies measuring biochemical indicator levels in blood and CSF.**

| **Trial（author）** | | **Year** | | **Location** | | | **Participants（ALSs）** | | | **Participants（controls）** | **Case ascertainment** | **age at baseline, mean (SD)** | | **% male** | | **measurements** |
| --- | --- | --- | --- | --- | --- | --- | --- | --- | --- | --- | --- | --- | --- | --- | --- | --- |
|  |  |  |  |  |  |  |  |  |  |  |  | ***ALSs*** | ***control*** | ***ALSs*** | ***controls*** |  |
| Wu, Y. | | 2020 | | China | | | 31 | | | 34 | Clinical | 55.13 ± 14.07 | 47.21 ± 17.72 | 64.5 | 44.12 | CSF glucose, CSF total protein, Qalb |
| Grunseich, Christopher | | 2020 | | USA | | | 22 | | | 1632 | Clinical | 42 | NA | 50 | 69.91 | Creatine kinase |
| Ito, D. | | 2019 | | Japan | | | 39 | | | 20 | Clinical | 66.4 ± 7.3 | 64.4 ± 6.9 | 64.1 | 65 | LDL-C, HDL-C, serum albumin, serum total protein, Creatine kinase |
| Chen, X. | | 2019 | | China | | | 571 | | | 571 | Clinical | 53.17 ± 11.72 | 53.17 ± 11.71 | 56.57 | 56.57 | Fasting blood glucose |
| Costa, J. | | 2019 | | Portugal | | | 26 | | | 10 | Clinical | 57.0 (49.1–64.3) | 64.6 (55.7–72.3) | 69.23 | 60 | CSF total protein |
| Ikenaka, Kensuke M. | | 2019 | | Japan | | | 53 | | | 20 | Clinical | 65.0 (58.5–71.5) | 62.0 (48.5–71.5) | 61.54 | 60 | CSF total protein |
| Qionghua Sun. | | 2019 | | China | | | 434 | | | 336 | Hospital | 55.33 ± 9.32 | 54.85 ± 14.26 | 64.75 | 53.57 | Serum ferritin, Serum transferrin, Serum iron, TIBC |
| Lombardi, V. | | 2019 | | UK | | | 53 | | | 73 | Clinical | 63 ± 14 | 60.04 ± 8.7 | 87.1 | 58.11 | Creatine kinase |
| Barros, Anab | | 2018 | | Brazil | | | 27 | | | 26 | Clinical | 56.0 ± 12.6 | 55.4 ± 12.5 | 48 | 42 | Total cholesterol, LDL-C, HDL-C, TG |
| Rossi, D. | | 2018 | | Italy | | | 190 | | | 82 | Clinical | 64 (57–71) | 64 (52–72) | 60.47 | 64.54 | CSF glucose, CSF total protein, Qalb |
| Kułakowska, A. | | 2018 | | Poland | | | 12 | | | 7 | Clinical | 57.5 ± 10.3 | 48.4 ± 15.3 | 25 | 28.57 | CSF total protein, Qalb |
| Yu, J. | | 2018 | | China | | | 24 | | | 38 | Clinical | 51.96 ± 7.57 | 53.32 ± 3.28 | 58.33 | 73.68 | Serum ferritin |
| Kim, S. M. | | 2017 | | Korean | | | 39 | | | 33 | Clinical | 53.8 ± 12.15 | 50.52 ± 16.51 | 52 | 48 | Total cholesterol |
| Delaye, J. B. | | 2017 | | France | | | 30 | | | 29 | ALS center | 66.5 ± 6.5 | 66.3 ± 6.4 | 50 | 51.8 | Total cholesterol, HDL-C |
| Zheng, Y. | | 2017 | | China | | | 54 | | | 46 | Hospital | 53.85 ± 4.98 | 51.26 ± 4.13 | 61.11 | 52.17 | Serum transferrin |
| Nagase, M. | | 2016 | | Japan | | | 26 | | | 55 | Clinical | 60.9 ± 11.2 | 60.1 ± 9.3 | 53.85 | 69.09 | Total cholesterol |
| Feneberg, E. | | 2016 | | German | | | 16 | | | 17 | Clinical | 56 (41–76) | 44 (18–88) | 62.5 | 29.41 | Qalb |
|  | |  | |  | | |  | | |  |  |  |  |  |  |  |
| Lu, C. H. | | 2016 | | UK | | | 95 | | | 88 | Clinical | 66.8 (58.6–72.4) | 59.1 (53.5–66.6) | 66.3 | 29.5 | Serum ferritin, creatine kinase |
| Henriques, A. | | 2015 | | France | | | 48 | | | 48 | 2 ALS centers | 54.9 (34.9–68.7) | 54.1 (31.7–66.6) | 62.5 | 62.5 | Total cholesterol, TG |
| Su, Xiaowei W. | | 2015 | | USA | | | 138 | | | 152 | NA | 62.1 (30.8–82.7) | 39.9 (20.0–81.0) | 63 | 32.2 | Serum ferritin |
| May, C. | | 2014 | | Hungary | | | 20 | | | 20 | Clinical | 60 (44-77) | 60 (43-76) | 50 | 50 | Total cholesterol, LDL-C, HDL-C, TG, Fasting blood glucose, Serum Albumin, Serum iron, Creatine kinase |
| Wuolikainen, A | | 2014 | | Sweden | | | 52 | | | 40 | Clinical | M: 60.0 ± 13.2  F: 57.5 ± 14.3 | M:59.7±12.2, F:63.8±6.4 | 55.77 | 52.5 | Total cholesterol, LDL-C, HDL-C, TG |
| Wills, Anne-Marie | | 2014 | | USA | | | 17 | | | 7 | Multiple ALS centers | HC/HC: 57.5 ± 15.4, HF/HC: 64.0 ± 6.9 | 63.2 ± 9.4 | 41.18 | 86 | Total cholesterol, LDL-C, HDL-C, Fasting blood glucose, Insulin |
| Rui Huang. | | 2014 | | China | | | 413 | | | 400 | Clinical | 51.8 ± 10.2 | 51.4 ± 13.1 | 58.4 | 59.5 | Total cholesterol, LDL-C, HDL-C, TG |
| Blasco, H | | 2014 | | France | | | 81 | | | 37 | Clinical | 63.9 ± 11.6 | 67.9 ± 14.0 | 59 | 54.1 | Fasting blood glucose, CSF glucose |
| Veyrat-Durebex, C. | | 2014 | | France | | | 104 | | | 145 | Clinical | 67.6 ± 9.6 | 68.4 ± 15.6 | 51.92 | 53.05 | Serum ferritin, Serum transferrin, Serum iron, TIBC, TSC % |
| Yang JW. | | 2013 | | Korea | | | 95 | | | 99 | Clinical | 54.14 ± 9.88 | 52.52 ± 9.02 | 63.2 | 64.6 | Total cholesterol, LDL-C, HDL-C, TG, Fasting blood glucose, Serum total protein |
| Ticozzi, N. | | 2013 | | Italy | | | 259 | | | 40 | Clinical | NA | NA | NA | NA | Qalb |
| Ikeda K. | | 2012 | | Japan | | | 92 | | | 92 | NA | 58.8 ± 12.7 | 59.2 ± 11.6 | 62 | 62 | Total cholesterol, LDL-C, HDL-C, TG, Serum ferritin, Serum transferrin |
| Nadjar, Yann. | | 2012 | | Paris | | | 694 | | | 297 | Clinical | 61.85 ± 12.00 | 48.99 ±13.01 | 51,92 | 58.33 | Serum ferritin, Serum transferrin, Serum iron, TSC % |
| Sutedja NA. | | 2011 | | Netherlands | | | 303 | | | 2100 | Clinical | 64 (range: 24-85) | M:61(range: 40-80) F:57 (range: 50-70) | 57 | 19.05 | Total cholesterol, LDL-C, HDL-C |
| Pradat, P. F. | | 2010 | | France | | | 21 | | | 21 | Clinical | 53.2 ± 12.7 | 53.1 ± 12.9 | 85.71 | 85.71 | Total cholesterol, LDL-C, HDL-C, TG, Fasting blood glucose, Insulin |
| Süssmuth, S. D. | | 2010 | | German | | | 105 | | | 12 | Clinical | 61 (24–83) | 56 (31–70) | 29.52 | 83.33 | CSF Total protein, Qalb |
|  | |  | |  | | |  | | |  |  |  |  |  |  |  |
| Mitchell, Ryan M. | | 2010 | | USA | | | 29 | | | 36 | Clinical | Wt/Wt: 61(48–75), H63D/Wt: 60(48–72) | Wt/Wt: 54 (47-75), H63D/Wt: 58 (50–70) | Wt/Wt:61.11, H63D/Wt: 63.64 | Wt/Wt: 63.16, H63D/Wt: 58.82 | Serum transferrin |
| Chio A. | | 2009 | | Italy | | | 658 | | | 658 | Multidisciplinary Centers | 62.9 ± 10.5 | 62.7 ± 11.3 | 52.3 | 52.3 | Total cholesterol, LDL-C, HDL-C, TG |
| Dupuis L. | | 2008 | | NA | | | 369 | | | 286 | Hospital | 57.5 ± 13.0 | NA | 56.5 | NA | Total cholesterol, LDL-C, HDL-C, TG, |
| Qureshi, M. | | 2008 | | USA | | | 30 | | | 30 | Clinical | 56.3 ± 13.0 | NA | 56.67 | 46.67 | Serum ferritin |
| Goodall, Emily F. | | 2008 | | UK | | | 60 | | | 44 | Hospital | 60 (54–68) | 59 (52–64) | 68.33 | 31.82 | Serum ferritin, Serum transferrin, Serum iron, TIBC, TSC % |
| Sohmiya, M. | | 2005 | | Japan | | | 20 | | | 20 | Clinical | 61.2 ± 9.5 | 62.1 ± 12.4 | 65 | NA | Total cholesterol |
| Yushchenko, M. | | 2000 | | German | | | 14 | | | 20 | Clinical | NA | NA | NA | NA | Qalb |
| Ludolph, A. C. | | 1992 | | German | | | 21 | | | 12 | Hospital | 59.7 ± 11.3 | 51.3 ± 9.2 | 52.38 | 75 | Fasting blood glucose |
| Harris, M. D. | | 1986 | | USA | | | 13 | | | 10 | Hospital | 51.9 ± 13.2 | 51.6 ± 9.9 | 84.62 | 90 | Fasting blood glucose, Qalb |
| Jockers-Wretou, E. | | 1985 | | Greece | | | 13 | | | 200 | Clinical | NA | NA | 84.62 | 56 | Creatine kinase |
| Reyes, E. T. | | 1984 | | NA | | | 10 | | | 15 | NA | 48 ± 4 | 30 ± 2 | 100 | 53.33 | Fasting blood glucose |
| Murai, A. | | 1983 | | Japan | | | 23 | | | 17 | NA | 49.9 ± 14.2 | 30.4 ± 6.8 | NA | 94.12 | Fasting blood glucose, Insulin |
| Moxley, R. T. | | 1983 | | USA | | | 10 | | | 11 | NA | 48.2 ± 5 | 25.5 ± 2 | 40 | 63.64 | Fasting blood glucose, Insulin |
|  |  | |  | |  |  | |  |  |  |  |  |  |  |  |  |

| Abbreviations: ALS, amyotrophic lateral sclerosis; CSF, cerebrospinal fluid; F, female; HC/HC, high-carbohydrate hypercaloric diet; HF/HC, high-fat hypercaloric diet; HDL, high-density lipoprotein. M: male; NA: not available; LDL: low-density lipoprotein. Qalb: CSF/serum albumin quotient SD: standard deviation TC: total cholesterol. TG: triglyceride. TIBC: total iron-binding capacity TSC: transferrin saturation coefficient Wt: wild type; H63D:H63D HFE variant  Supplementary Table 2: Characteristics and summary statistics of studies showing the association of serum ferritin with survival in ALS patients.   \| ***Tria（author）*** \| ***Trial（year）*** \| ***Location*** \| ***Participants（ALSs）*** \| ***Group A*** \| ***Number of group A*** \| ***Group B*** \| ***Number of group B*** \| ***HR*** \| ***Low 95%CI*** \| ***High 95%CI*** \| ***P value*** \| \| --- \| --- \| --- \| --- \| --- \| --- \| --- \| --- \| --- \| --- \| --- \| --- \| \| *Qionghua Sun* \| 2019 \| China \| 149 \| high ferritin (>190 mg/L) \| 74 \| low ferritin (<190 mg/L) \| 75 \| 1.73 \| 1.15 \| 2.62 \| 0.01 \| \| *Lu, C. H.* \| 2016 \| UK \| 95 \| Not given \| Not given \| Not given \| Not given \| 1.74 \| 1.26 \| 2.41 \| 0.001 \| \| *Su, Xiaowei W.* \| 2015 \| USA \| 87 \| high ferritin (>=202 mg/L) \| 44 \| low ferritin (<202 mg/L) \| 43 \| 1.17 \| 0.72 \| 1.89 \| 0.778 \| \| *Su, Xiaowei W.* \| 2015 \| USA \| 51 \| high ferritin (>=123 mg/L) \| 26 \| low ferritin (<123 mg/L) \| 25 \| 0.73 \| 0.39 \| 1.4 \| 0.36 \| \| *Patin, F.* \| 2015 \| France \| 216 \| Not given \| Not given \| Not given \| Not given \| 68.746 \| 4.186 \| 854.3 \| 0.0048 \| \| *Nadjar, Yann* \| 2012 \| France \| 594 \| high ferritin (>156 mg/L) \| 297 \| low ferritin (<156 mg/L) \| 297 \| 1.19 \| 1.02 \| 1.37 \| 0.013 \|   Supplementary Table 3: Characteristics and summary statistics of studies showing the association of creatine kinase with survival in ALS patients.   \| ***Tria（author）*** \| ***Trial（year）*** \| ***Location*** \| ***Participants（ALSs）*** \| ***Group A*** \| ***Number of group A*** \| ***Group B*** \| ***Number of group B*** \| ***HR*** \| ***Low 95%CI*** \| ***High 95%CI*** \| ***P value*** \| \| --- \| --- \| --- \| --- \| --- \| --- \| --- \| --- \| --- \| --- \| --- \| --- \| \| *Ikenaka, Kensuke M.* \| 2019 \| Japan \| 25 \| Not given \| Not given \| Not given \| Not given \| 1.002 \| 1 \| 1.004 \| 0.094 \| \| *Tai, H.* \| 2017 \| China \| 185 \| Not given \| High CK \| Not given \| Norm CK \| 0.457 \| 0.221 \| 0.947 \| 0.035 \| \| *Rafiq, M. K.* \| 2016 \| Europe \| 512 \| Not given \| Not given \| Not given \| Not given \| 0.74 \| 0.59 \| 0.93 \| 0.013 \| \| *Gibson, S. B.* \| 2015 \| USA \| 80 \| High CK（>200） \| Not given \| Low CK(<=200) \| Not given \| 3.57 \| 1.84 \| 7.27 \| <0.001 \| |  |
| --- | --- | --- | --- | --- | --- | --- | --- | --- | --- | --- | --- | --- | --- | --- | --- | --- | --- | --- | --- | --- | --- | --- | --- | --- | --- | --- | --- | --- | --- | --- | --- | --- | --- | --- | --- | --- | --- | --- | --- | --- | --- | --- | --- | --- | --- | --- | --- | --- | --- | --- | --- | --- | --- | --- | --- | --- | --- | --- | --- | --- | --- | --- | --- | --- | --- | --- | --- | --- | --- | --- | --- | --- | --- | --- | --- | --- | --- | --- | --- | --- | --- | --- | --- | --- | --- | --- | --- | --- | --- | --- | --- | --- | --- | --- | --- | --- | --- | --- | --- | --- | --- | --- | --- | --- | --- | --- | --- | --- | --- | --- | --- | --- | --- | --- | --- | --- | --- | --- | --- | --- | --- | --- | --- | --- | --- | --- | --- | --- | --- | --- | --- | --- | --- | --- | --- | --- | --- | --- | --- | --- | --- | --- | --- | --- | --- |
