# Supplementary material for: Aberrations of biochemical indicators in amyotrophic lateral sclerosis: a systematic review and meta-analysis
Source: Transl Neurodegener. 2021 Jan 8;10:3. doi: 10.1186/s40035-020-00228-9 (PMC7792103; doi:10.1186/s40035-020-00228-9)

Supplementary figure 1: Forest plot showing separate analysis of total cholesterol, low-density lipoprotein cholesterol, triglyceride, fasting blood glucose, fasting insulin, CSF glucose, CSF total protein, Qalb, serum albumin, serum total protein, serum ferritin, serum transferrin, serum iron, total iron binding capacity, transferrin saturation coefficient and creatine kinase based on ethnicity, respectively. Individual study values and their corresponding 95% confidence intervals are indicated by a short solid line. The weighted mean difference (WMD) and 95% confidence interval are indicated by a diamond.


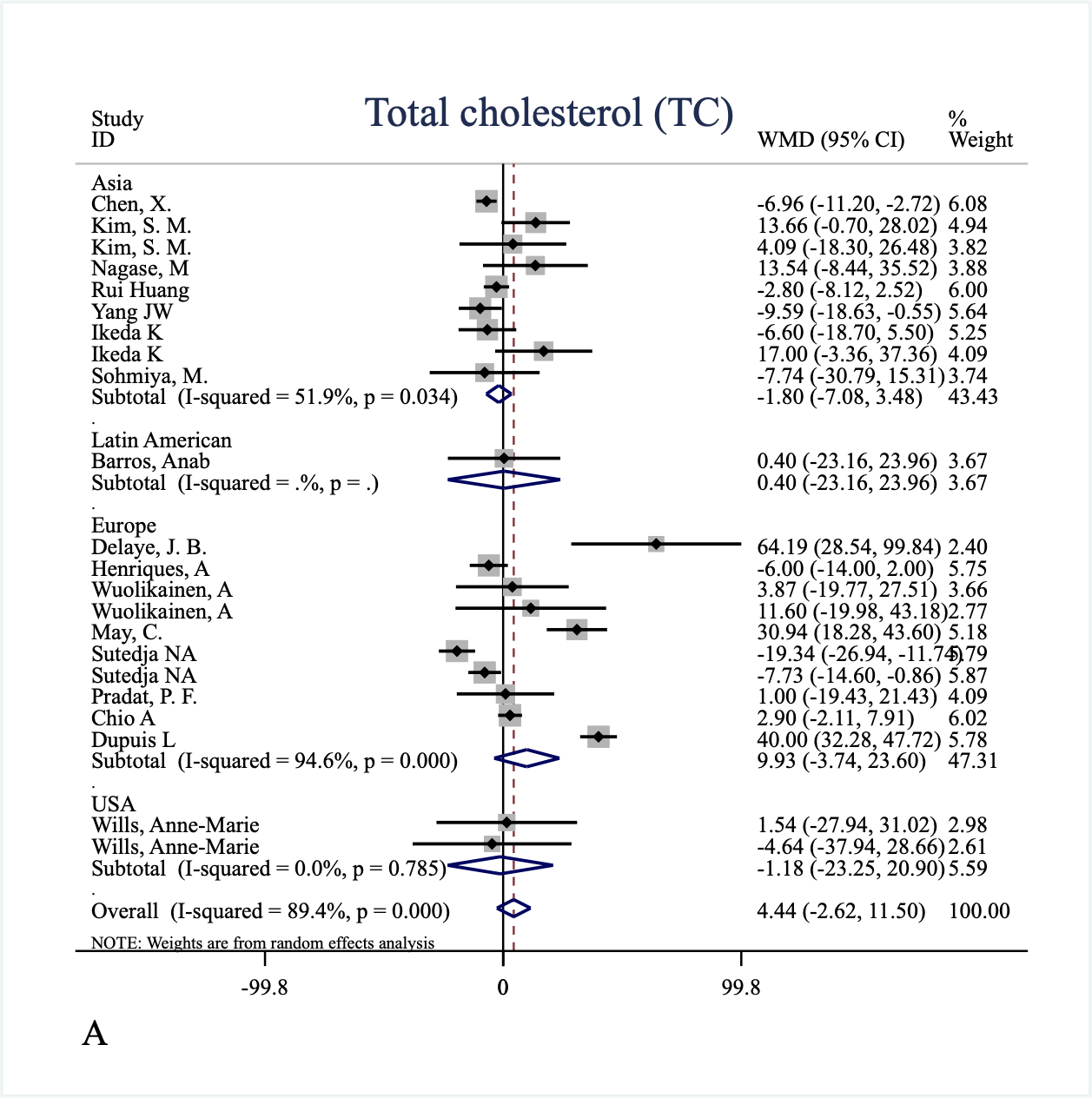


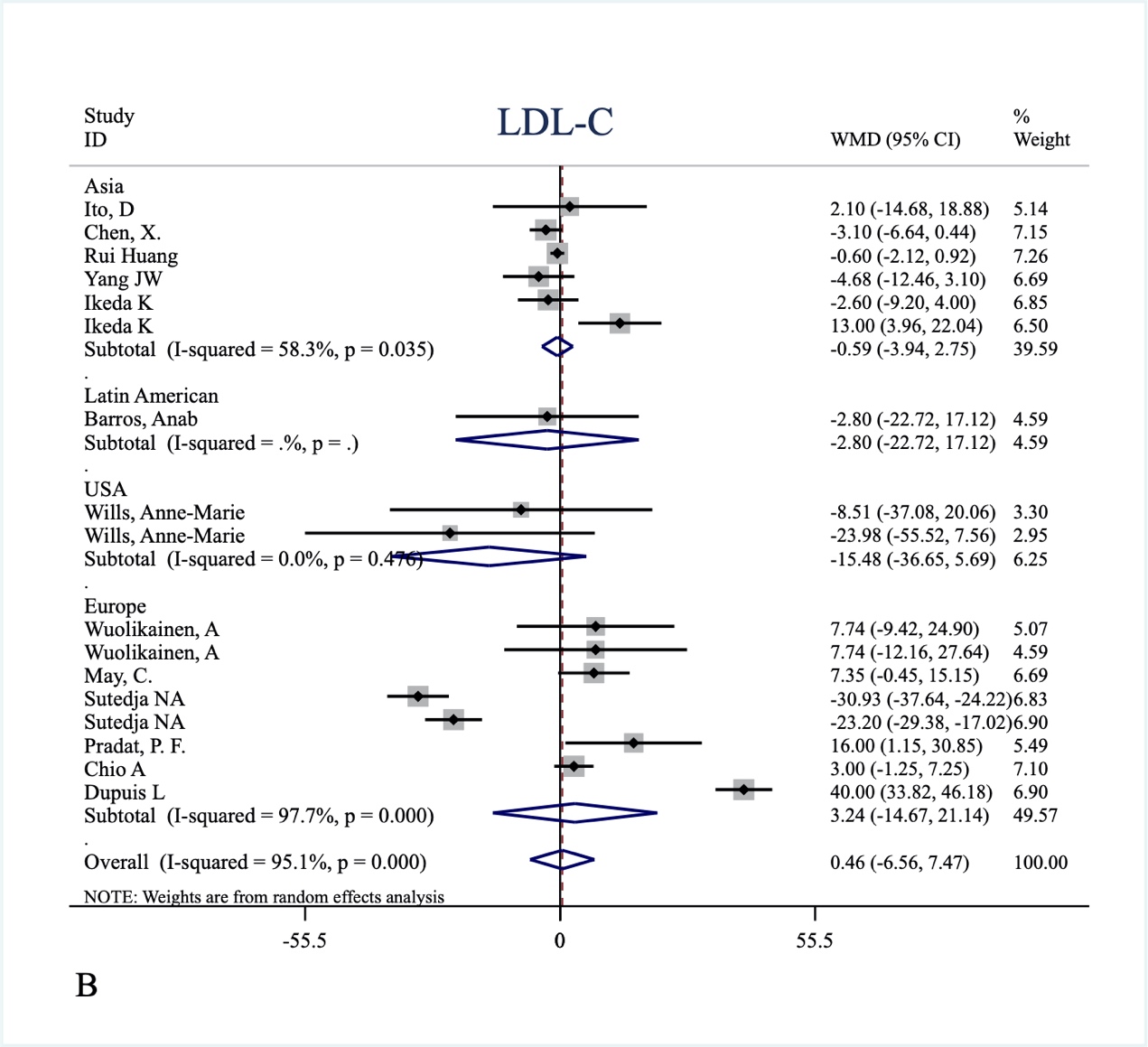


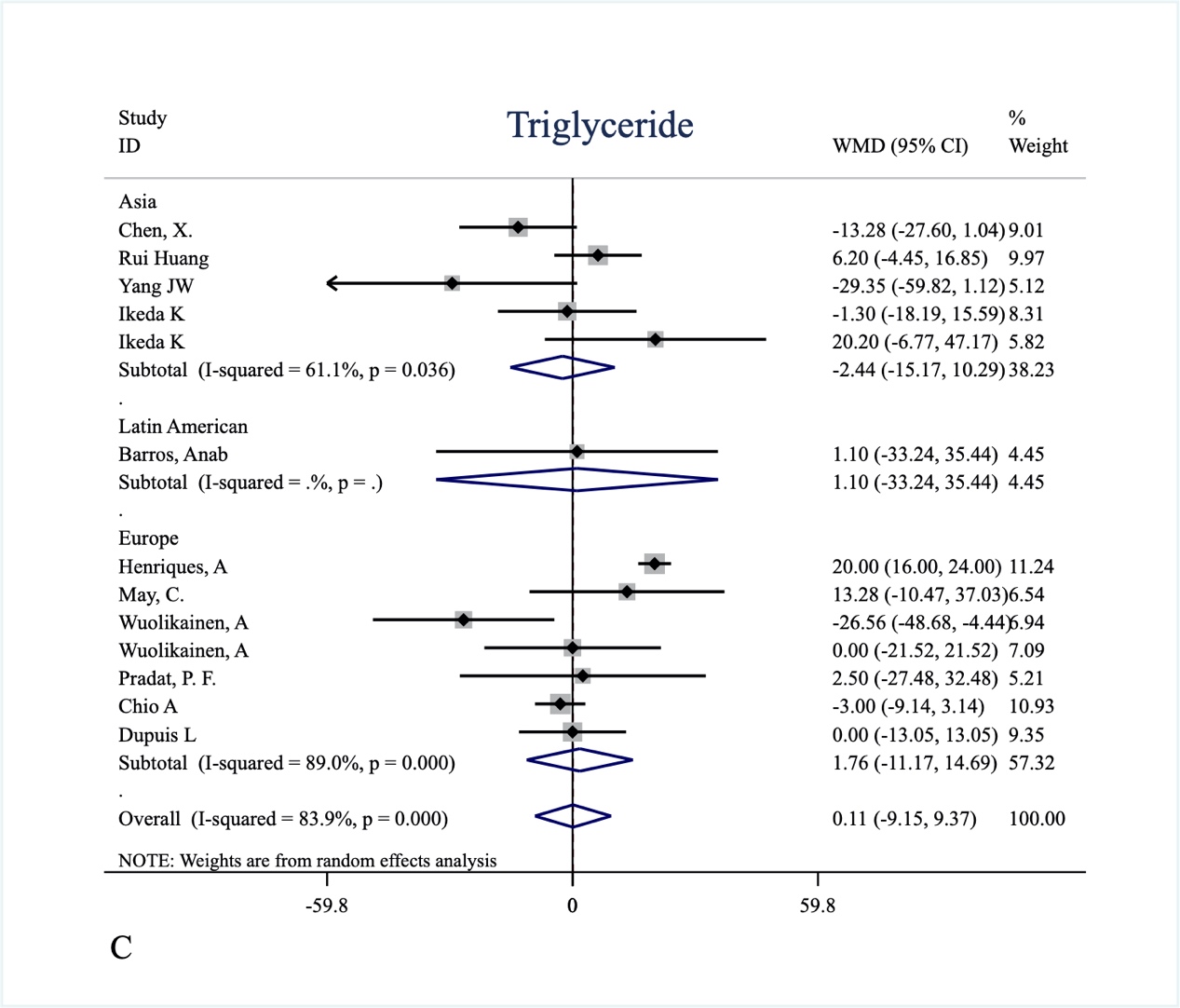


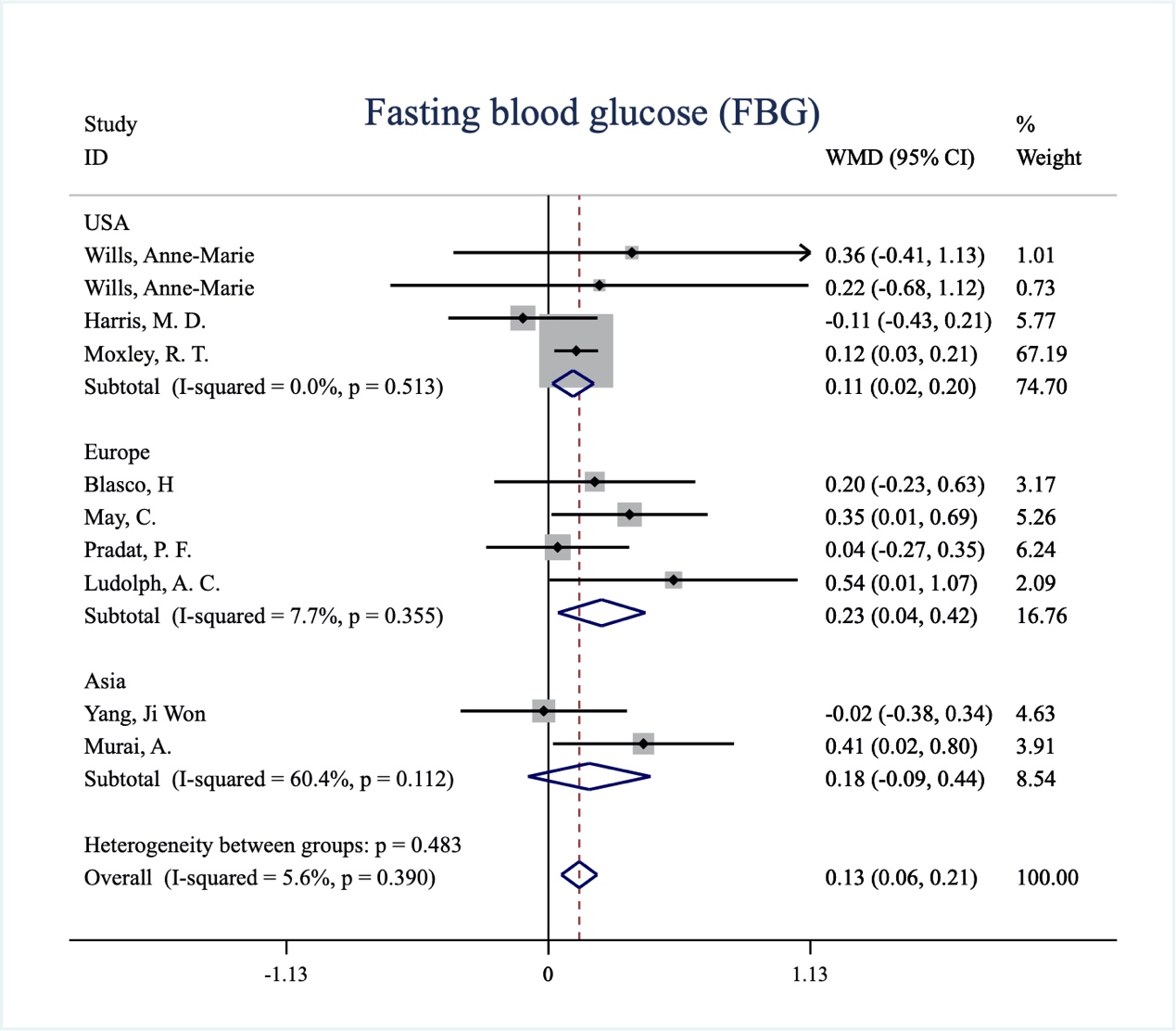


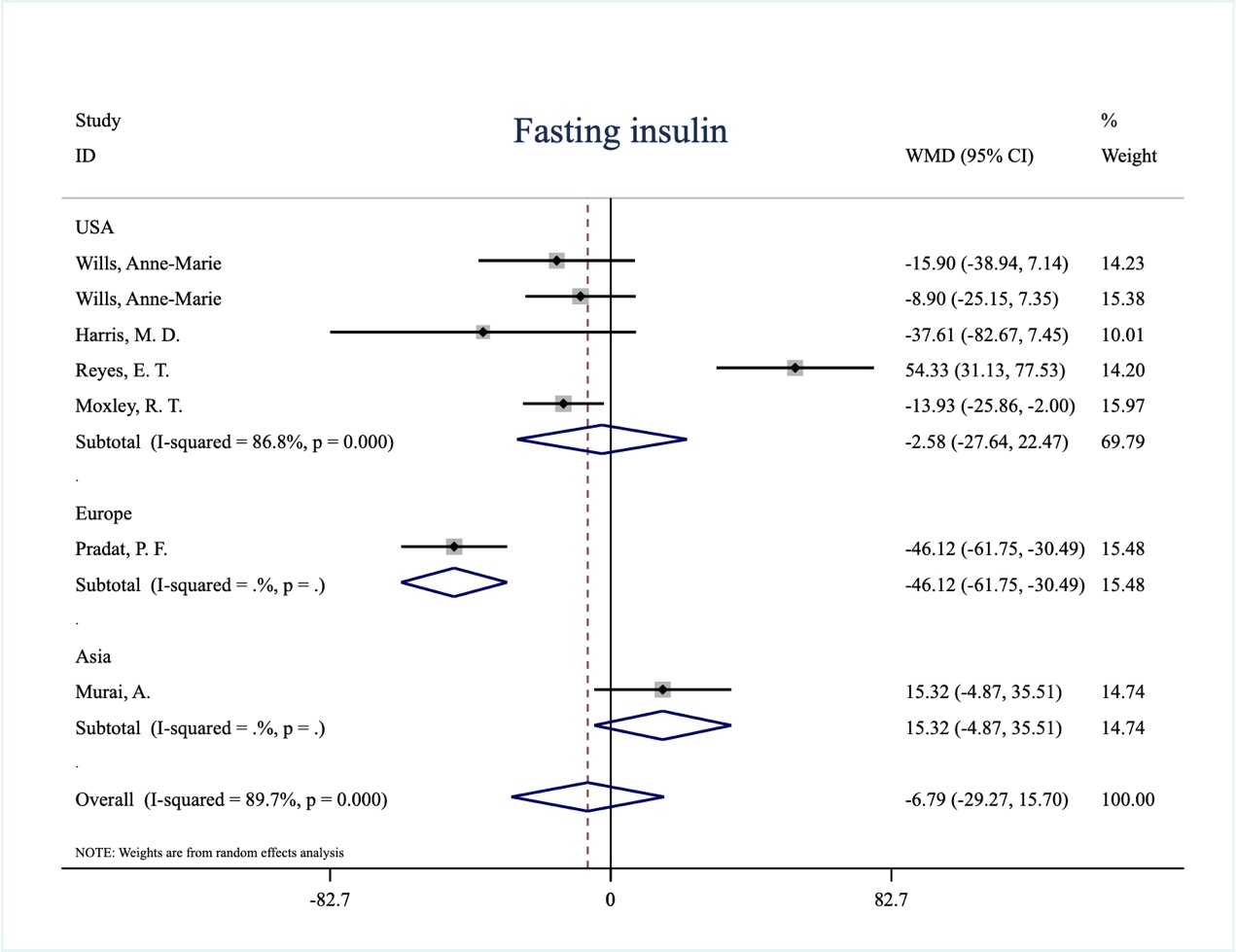


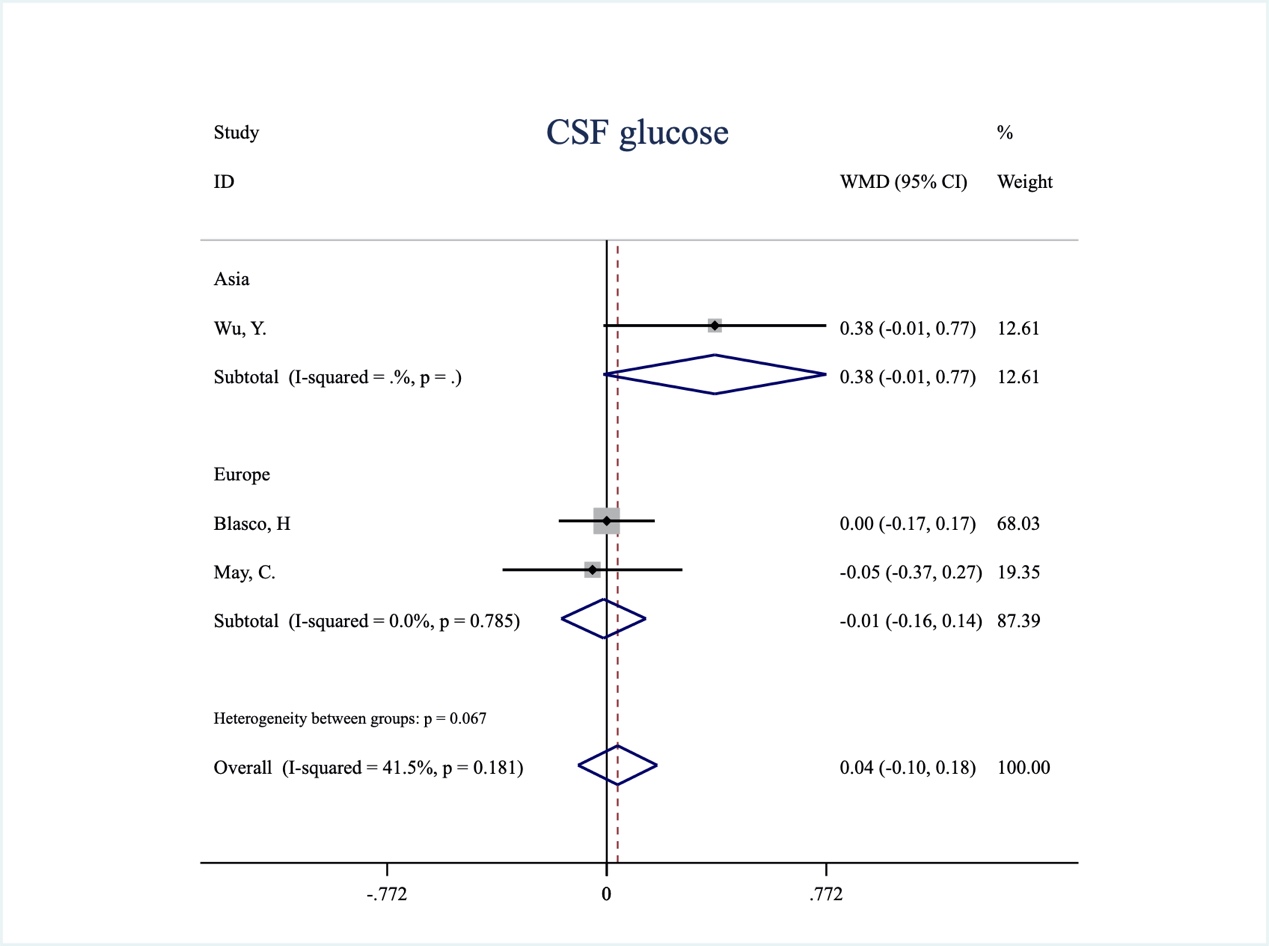


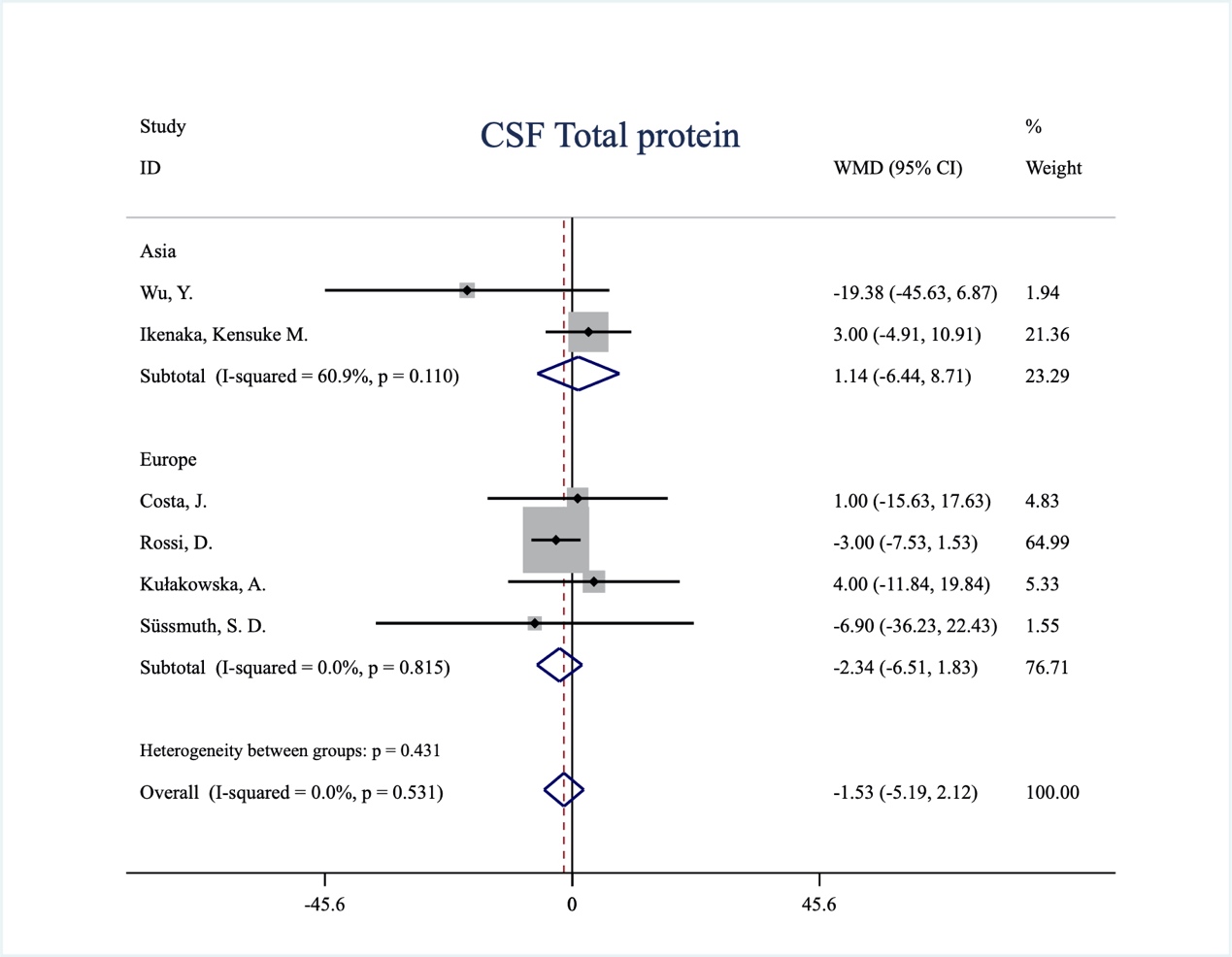


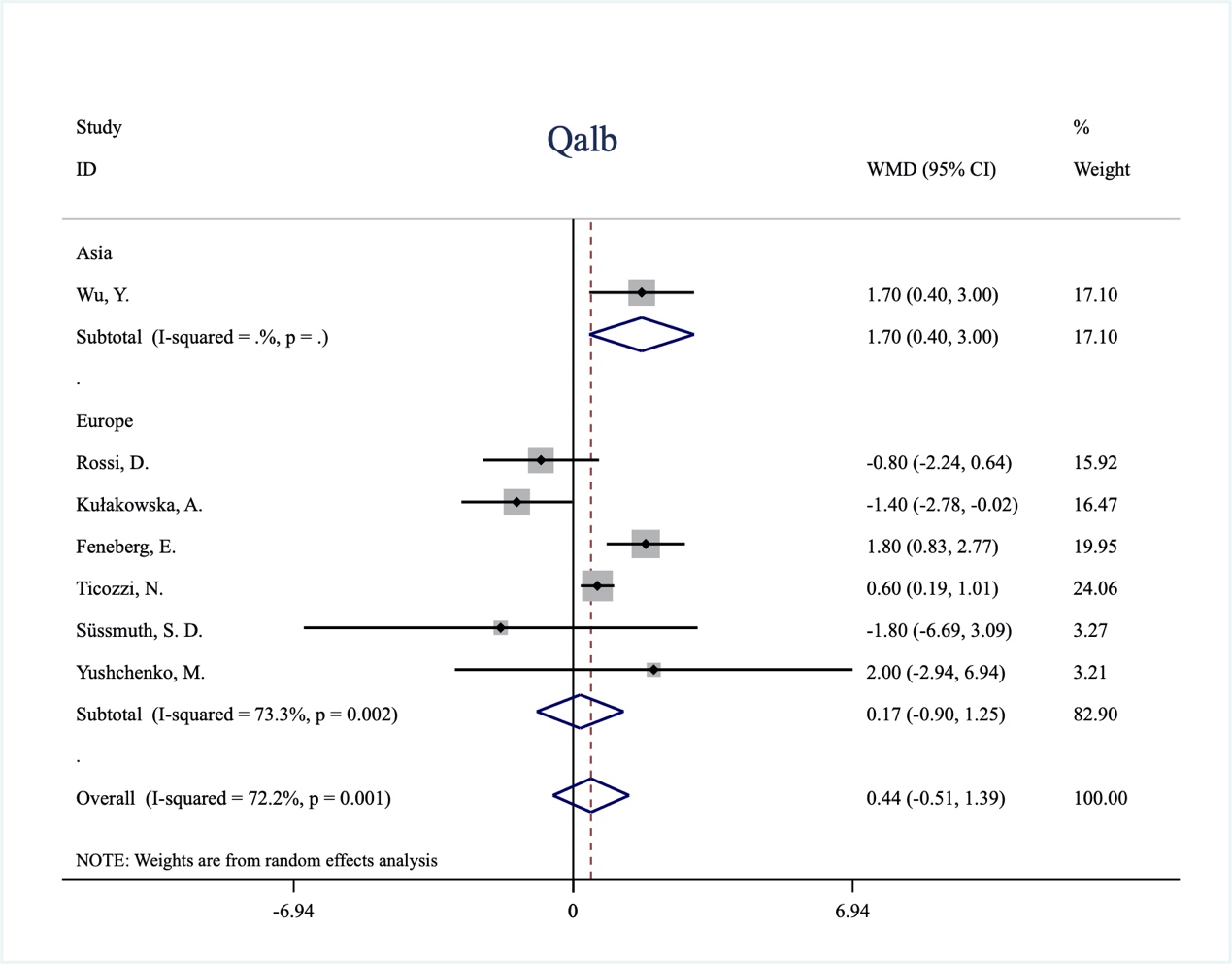


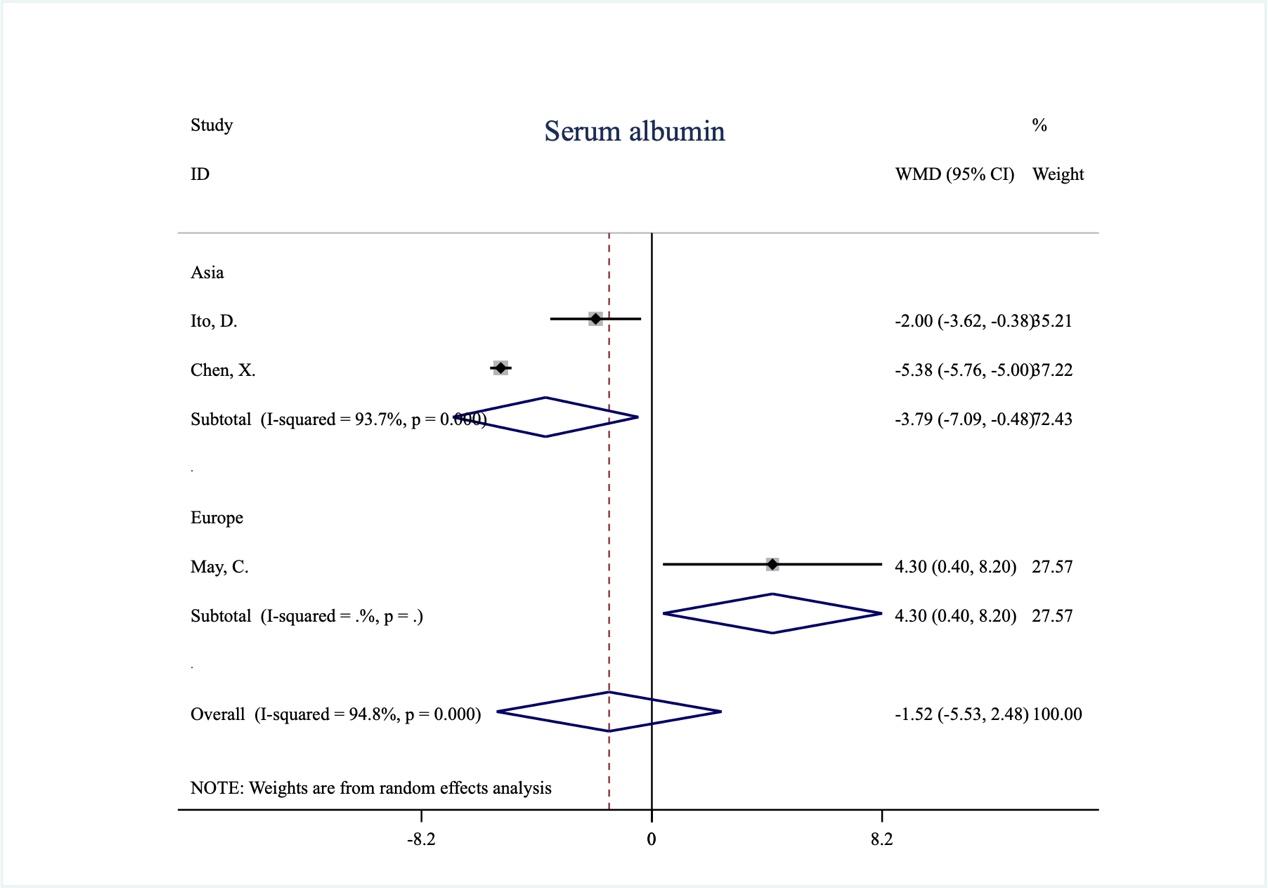


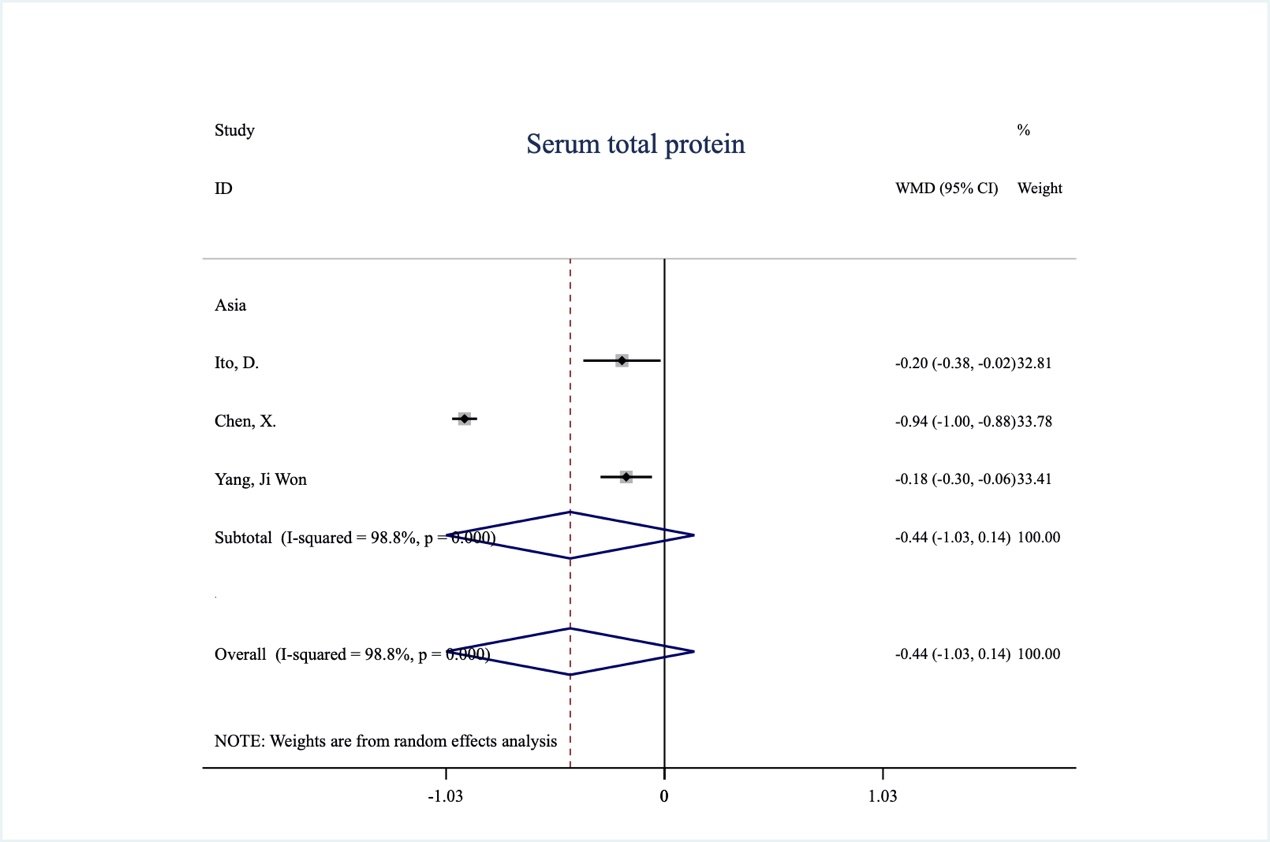


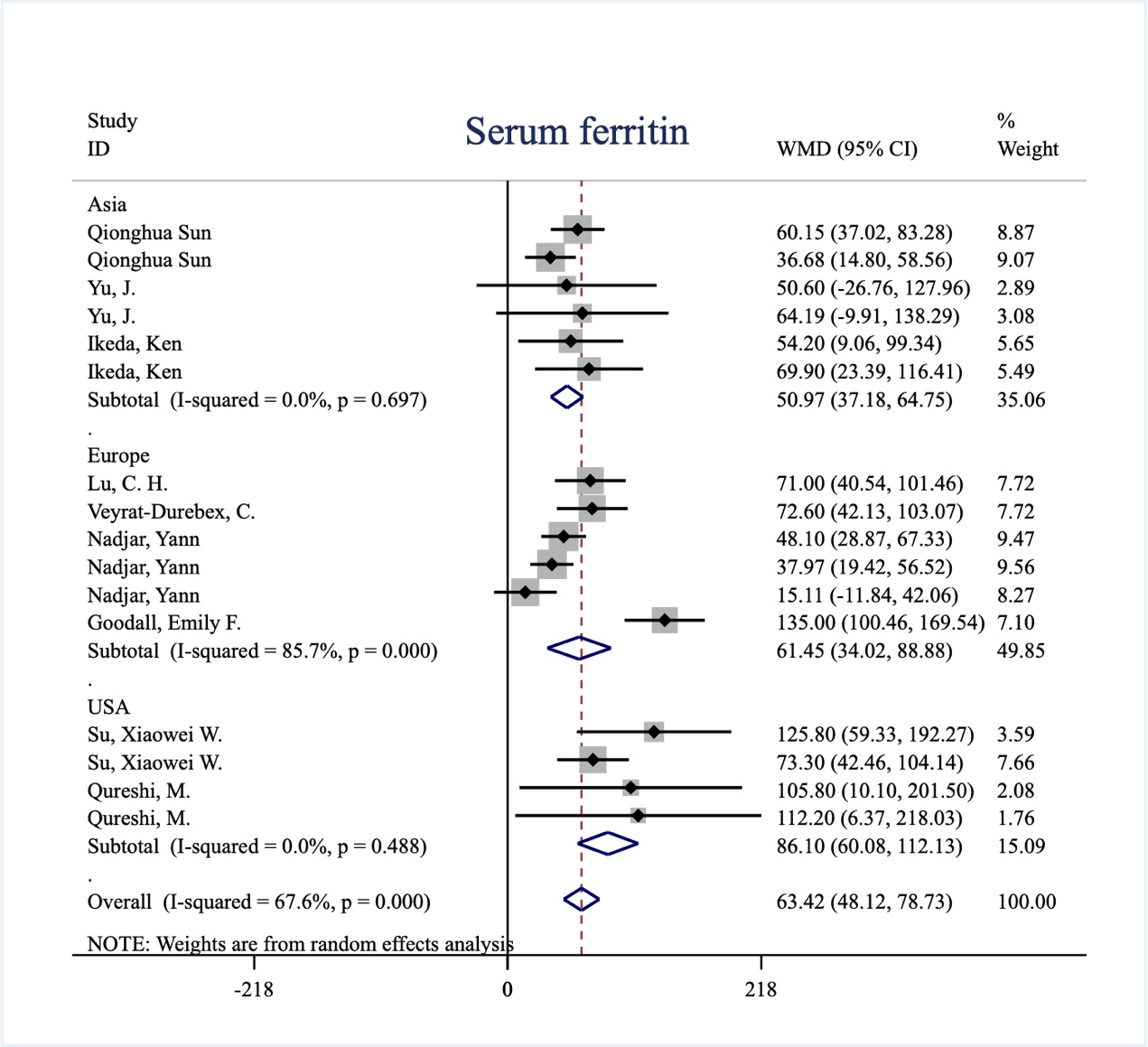


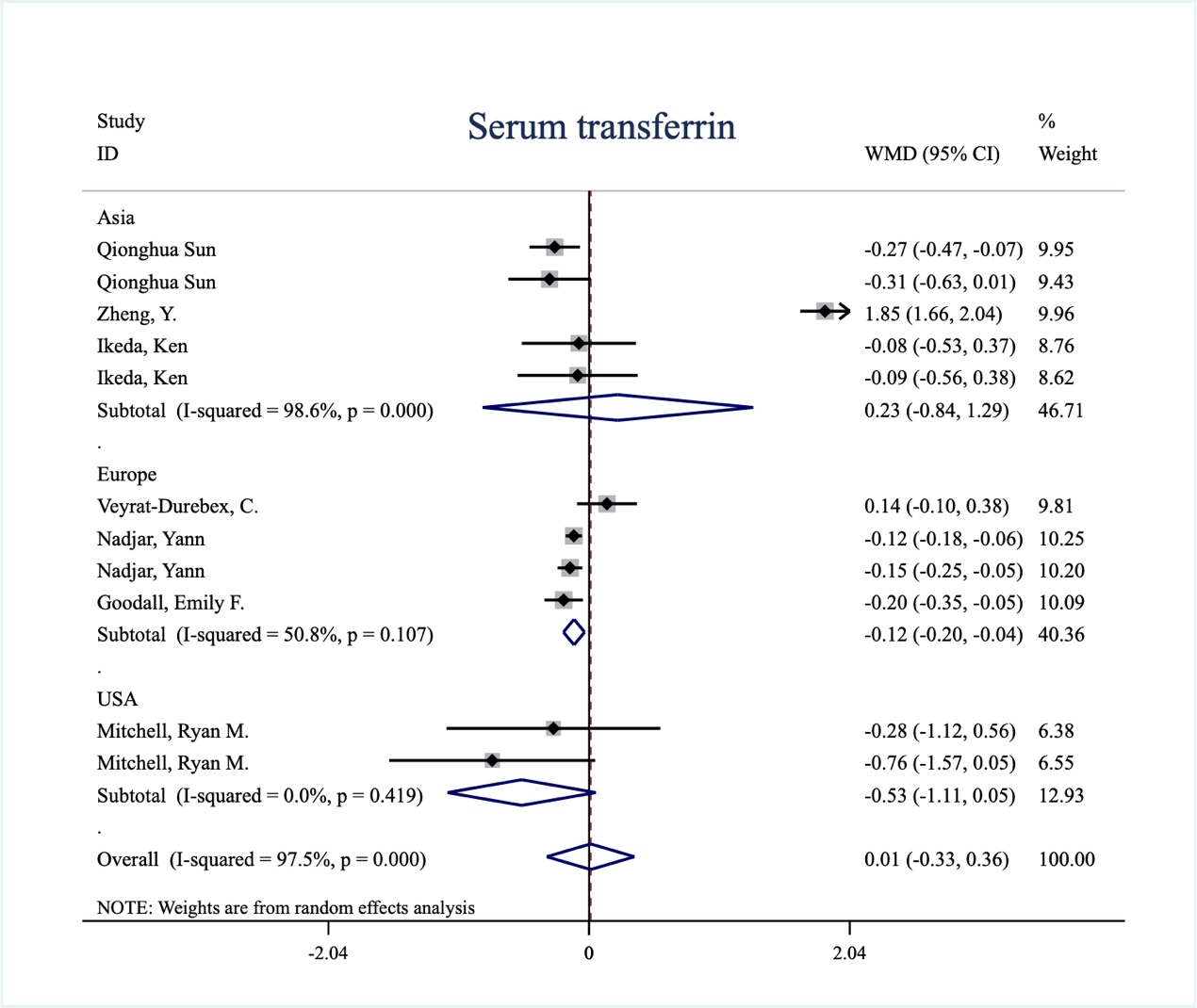


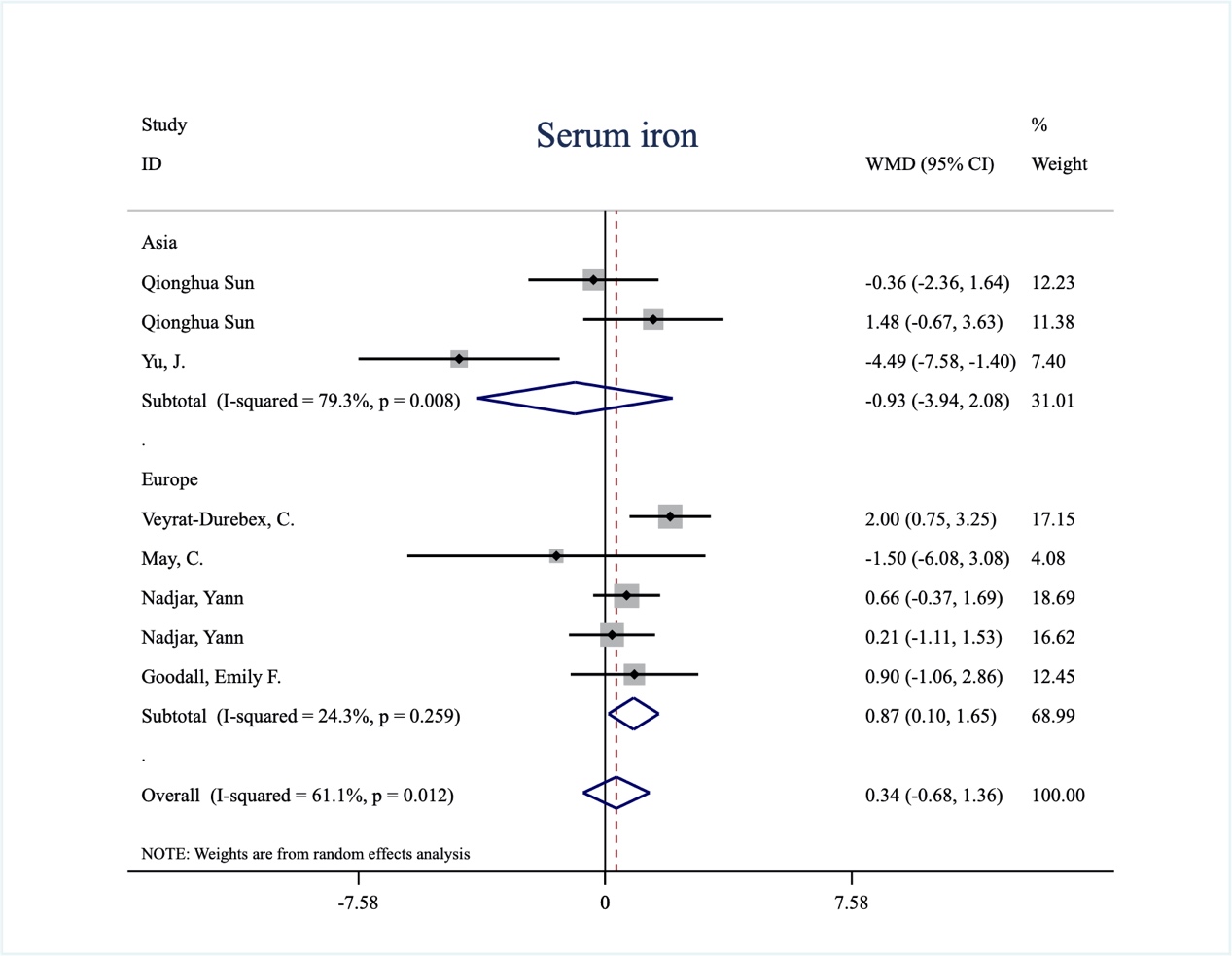


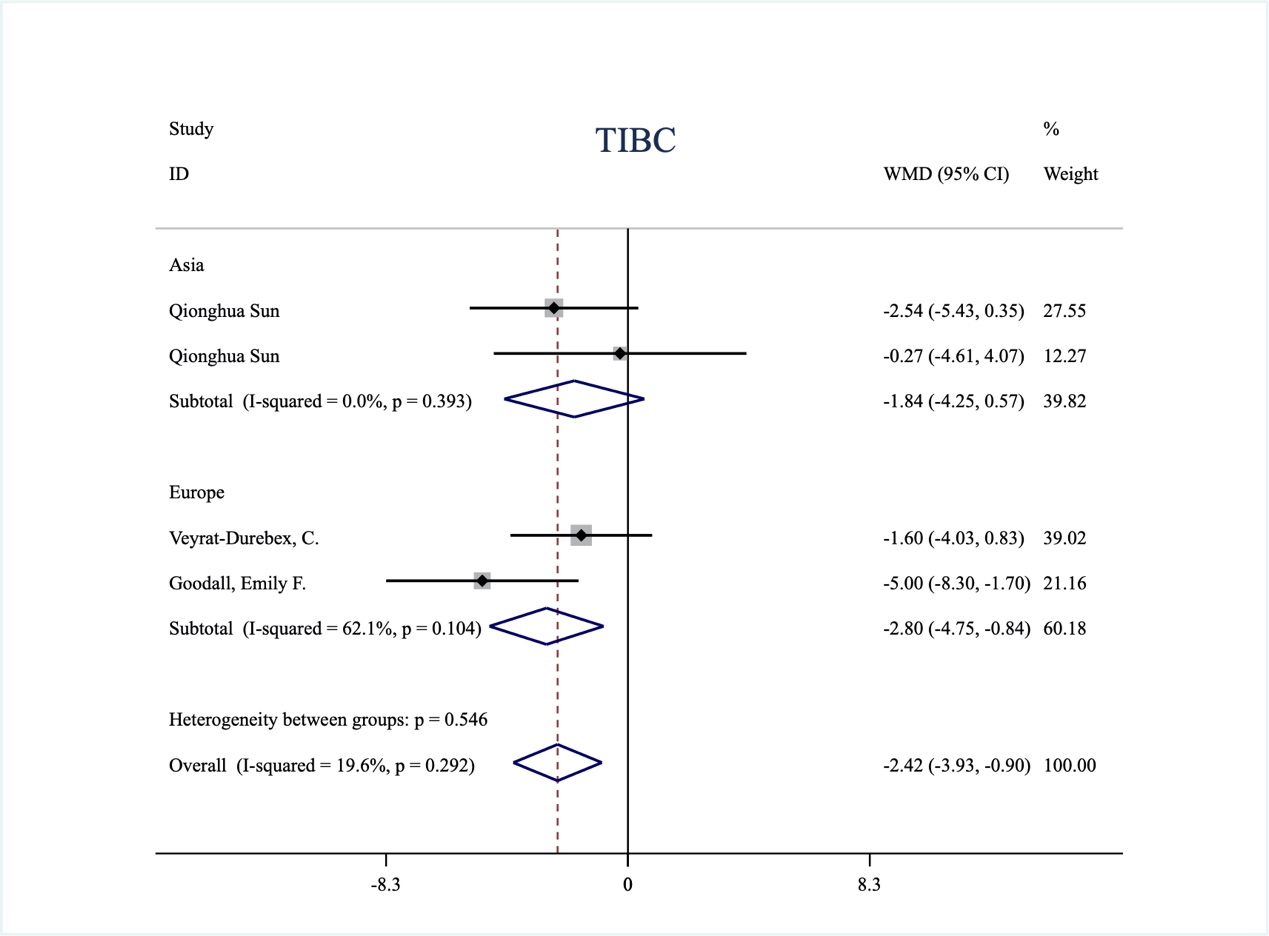


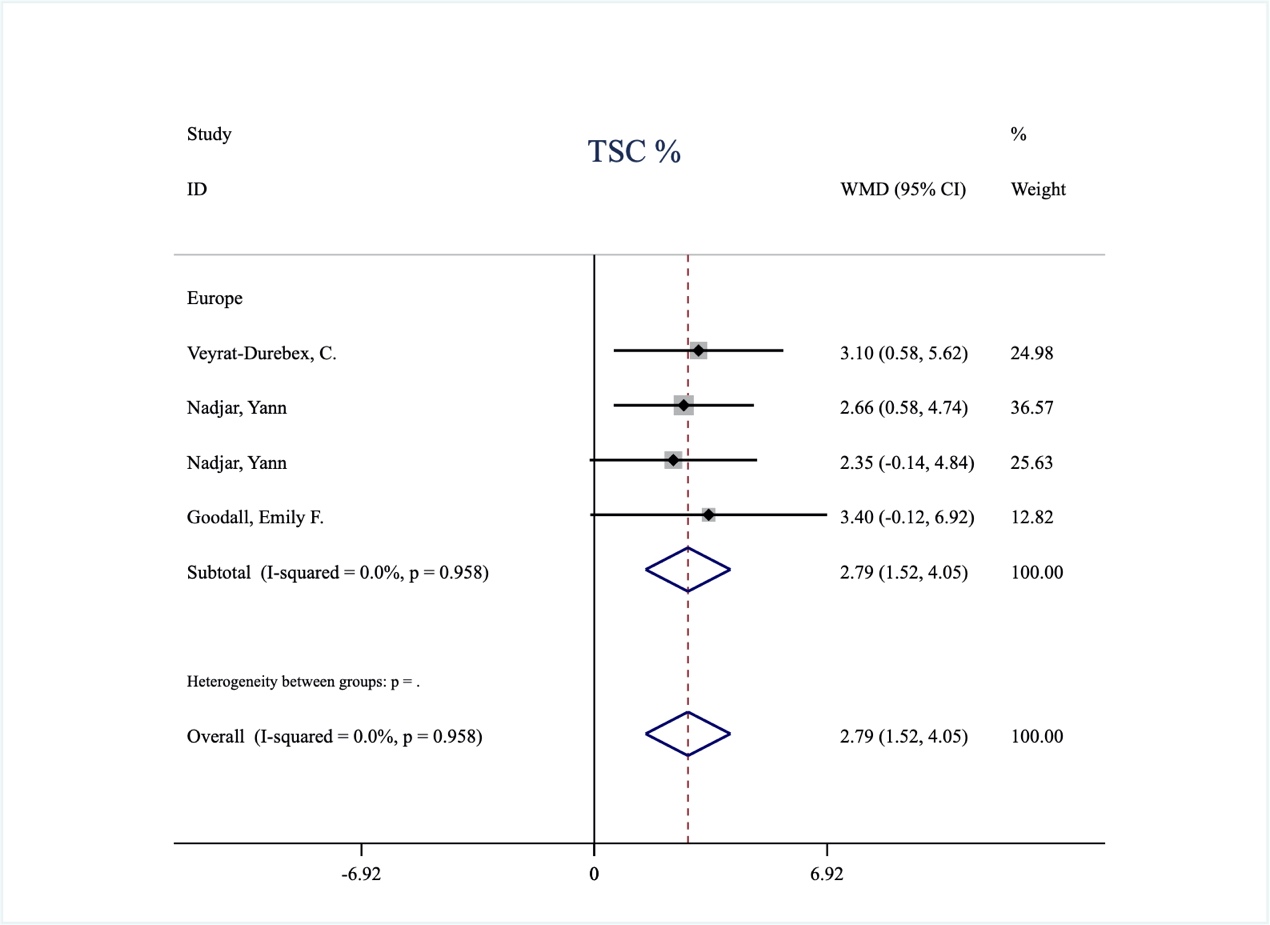


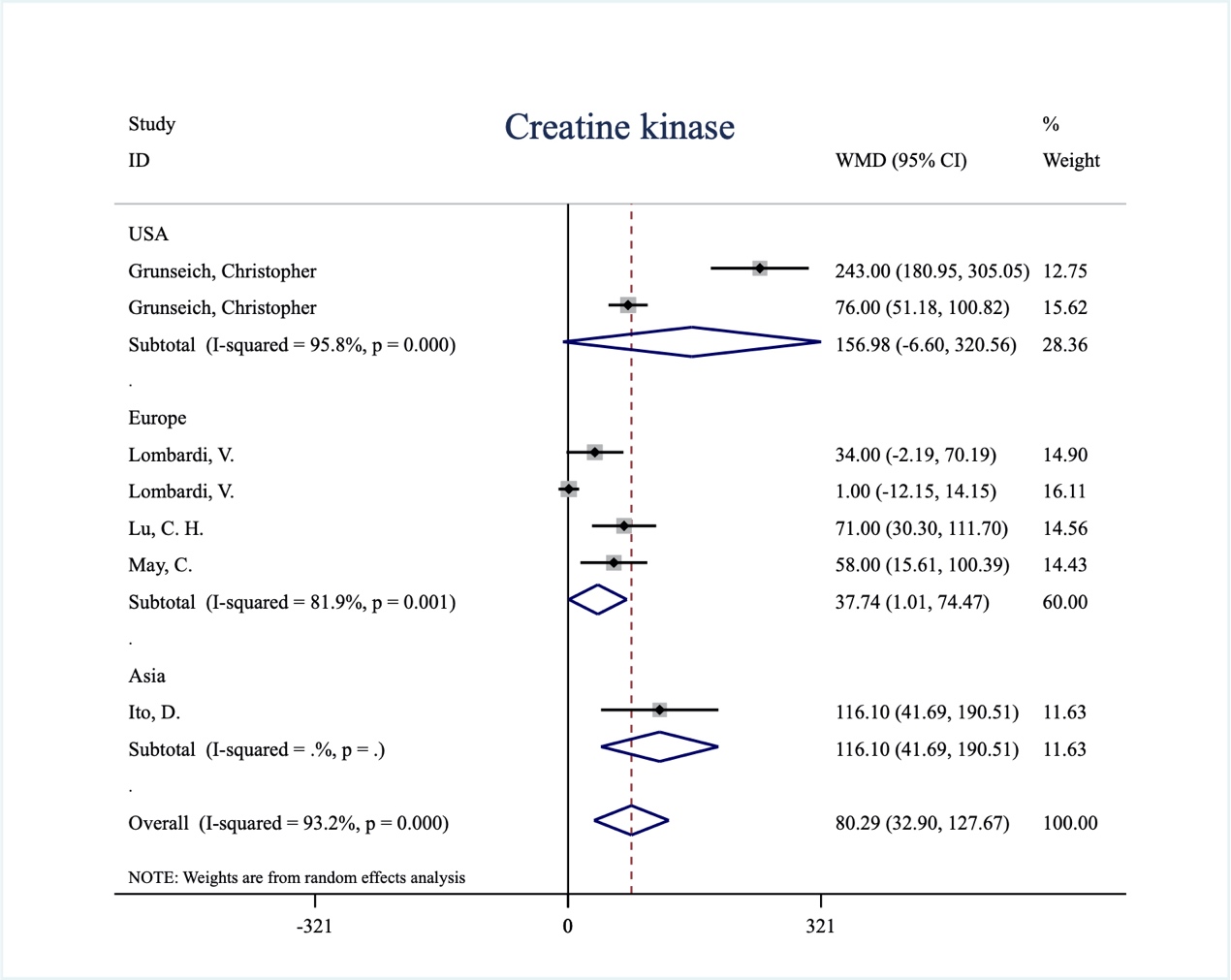

Supplement: Supplementary file 2 — Additional file 2: Fig. S1. Forest plot showing separate analysis of total cholesterol, low-density lipoprotein cholesterol, triglyceride, fasting blood glucose, fasting insulin, CSF glucose, CSF total protein, Qalb, serum albumin, serum total protein, serum ferritin, serum transferrin, serum iron, total iron binding capacity, transferrin saturation coefficient and creatine kinase based on ethnicity, respectively. Values and the corresponding 95% confidence intervals of individual studies are indicated by short solid lines. The weighted mean difference (WMD) and 95% confidence intervals are indicated by diamonds. [file 40035_2020_228_MOESM2_ESM.docx]
